# Ty1-*copia* elements reveal diverse insertion sites linked to polymorphisms among flax (*Linum usitatissimum* L.) accessions

**DOI:** 10.1186/s12864-016-3337-3

**Published:** 2016-12-07

**Authors:** Leonardo Galindo-González, Corinne Mhiri, Marie-Angèle Grandbastien, Michael K. Deyholos

**Affiliations:** 1 Department of Biological Sciences, University of Alberta, Edmonton, AB T6G 2E9 Canada; 2 Institut Jean-Pierre Bourgin, INRA, AgroParisTech, CNRS, Université Paris-Saclay, RD10, 78026 Versailles Cedex, France; 3 Department of Biology, University of British Columbia, Okanagan campus, Kelowna, BC V1V 1V7 Canada

**Keywords:** Ty1*-copia*, Transposable elements, Flax, Cultivars, Sequence-Specific Amplification Polymorphism (SSAP)

## Abstract

**Background:**

Initial characterization of the flax genome showed that Ty1*-copia* retrotransposons are abundant, with several members being recently inserted, and in close association with genes. Recent insertions indicate a potential for ongoing transpositional activity that can create genomic diversity among accessions, cultivars or varieties. The polymorphisms generated constitute a good source of molecular markers that may be associated with phenotype if the insertions alter gene activity. Flax, where accessions are bred mainly for seed nutritional properties or for fibers, constitutes a good model for studying the relationship of transpositional activity with diversification and breeding. In this study, we estimated copy number and used a type of transposon display known as Sequence-Specific Amplification Polymorphisms (SSAPs), to characterize six families of Ty1-*copia* elements across 14 flax accessions. Polymorphic insertion sites were sequenced to find insertions that could potentially alter gene expression, and a preliminary test was performed with selected genes bearing transposable element (TE) insertions.

**Results:**

Quantification of six families of Ty1*-copia* elements indicated different abundances among TE families and between flax accessions, which suggested diverse transpositional histories. SSAPs showed a high level of polymorphism in most of the evaluated retrotransposon families, with a trend towards higher levels of polymorphism in low-copy number families. Ty1-*copia* insertion polymorphisms among cultivars allowed a general distinction between oil and fiber types, and between spring and winter types, demonstrating their utility in diversity studies. Characterization of polymorphic insertions revealed an overwhelming association with genes, with insertions disrupting exons, introns or within 1 kb of coding regions. A preliminary test on the potential transcriptional disruption by TEs of four selected genes evaluated in three different tissues, showed one case of significant impact of the insertion on gene expression.

**Conclusions:**

We demonstrated that specific Ty1*-copia* families have been active since breeding commenced in flax. The retrotransposon-derived polymorphism can be used to separate flax types, and the close association of many insertions with genes defines a good source of potential mutations that could be associated with phenotypic changes, resulting in diversification processes.

**Electronic supplementary material:**

The online version of this article (doi:10.1186/s12864-016-3337-3) contains supplementary material, which is available to authorized users.

## Background

Transposable elements (TEs) are DNA fragments that can move between genomic locations using a cut and paste mechanism (DNA transposons), or a copy and paste mechanism via an RNA intermediate (retrotransposons). Transposition can result in alterations of gene expression and diversification between individuals, populations and species. TEs are commonly activated upon stresses that include tissue culture, wounding, microbial elicitors and pathogen attack [1–8]. Polyploidization (whether spontaneous or induced) also mobilizes transposable elements, resulting in genome restructuring, and genetic and epigenetic effects on gene activity (reviewed in [9]). Selective breeding can also affect TE activity. For example, in vegetatively propagated grape clones, TE insertional polymorphisms constitute the largest class of mutations [10]. Genetic diversity associated with TE polymorphisms has been commonly explored in plant varieties and species such as pepper and tomato [2, 11], barley [12], strawberry [13], coffee [14], blue agave [15] and cashew [16].

We previously showed that more than 20% of the flax (*Linum usitatissimum*) genome is made of TEs [17, 18]. The main group represented in the genome are LTR (Long Terminal Repeat) retrotransposons, from which the Ty1*-copia* elements are the most abundant. Ty1-*copia* have five main domains encoding proteins required for the retrotransposition cycle: group-specific antigen (GAG), protease (PR), integrase (INT), reverse transcriptase (RT) and ribonuclease H (RH). Because of their retrotransposition mechanism, LTRs are identical at the time of TE insertion [19], and thus sequence variations in them can be used as a molecular clock of insertion. LTRs act as promoter sequences since they contain *cis-*acting elements that respond to different stress elicitors [20–25]. Ty1-*copia* elements can spread randomly throughout the genome and are more often associated with genes than Ty3-*gypsy* elements [17, 26, 27]. Therefore, Ty1-*copia* elements can alter gene regulation [28–30], promote transduction events of one or more genes to other genomic locations [31–33], or result in epigenetic gene silencing [34, 35]. Additionally, they can also fall inside genes disrupting gene function or altering splice patterns [36–39].

Numerous Ty1-*copia* members have been recently inserted in flax, as inferred from their LTR similarity and gene domain conservation (at least 83 Ty1-*copia* elements have 100% LTR similarity) [17]. Furthermore, Ty1*-copia* elements have had increasing activity in the flax genome starting five million years ago [17]. These observations indicate that Ty1*-copia* elements could generate polymorphisms among closely related flax cultivars.

Flax is a valuable source of bioproducts derived from the seed (i.e. linseed) and stem fiber [40]. Its breeding for either seed or fiber traits in diverse climates has resulted in diverse cultivars and an array of agrobotanic characteristics that have been artificially selected [40]. While flax grown for human consumption (seeds are used for nutrition but also for oil derived industrial products), is the same species as the flax grown mainly to manufacture linen, they represent two different flax types (oil and fiber) and the products are usually obtained from cultivars (or accessions) that have been bred to have mainly one of the two characteristics [40]. Additionally, flax is a summer annual crop in temperate climates and is usually sown during spring, but winter cultivars have been bred that can be sown in the autumn in milder climates. Flax is therefore an interesting system for studying the relationship of TEs to continuous and divergent selection practices.

The current study aims to uncover the impact of specific Ty1*-copia* retrotransposon families on diversification of flax cultivars. We measured the level of polymorphism among a set of flax cultivars, and analyzed their relationship using a TE-based marker system. Since TE insertions within genes are more likely to interfere with gene function, we characterized the nature of target sequences of polymorphic insertions to find out if they were closely associated with genes, and measured the effect of retrotransposon insertion on transcript expression in selected genes.

Several strategies have been devised to find TE insertional polymorphisms (reviewed in [41]). Previously, Inter-Retrotransposon Amplified Polymorphism (IRAP) was used to study flax cultivars and species [42]. Here we used a type of transposon display (TD), known as Sequence-Specific Amplification Polymorphism (SSAP) [43, 44], to evaluate Ty1*-copia* retrotransposon insertions in 14 flax accessions of either oil or fiber types, and spring or winter types. In SSAP, TEs and flanking DNA are preferentially amplified using a PCR primer that anneals to a sequence specific to a particular TE family (usually an LTR), and a second primer that anneals to an adaptor ligated to a restriction enzyme site. Our study showed that families of flax Ty1-*copia* TEs have high levels of polymorphism between cultivars, indicating recent activity since organized breeding commenced in the last century. While the copy number of each family did not vary greatly between cultivars, some families of TEs were consistently more abundant than others across multiple cultivars. Analysis of sequence insertion sites demonstrated that many of these Ty1-*copia* elements inserted within or in close proximity to genes. Finally, we found one case where an insertion of a TE in an exon of a Laccase gene decreased gene expression in roots. Our study demonstrates that TEs from the Ty1-*copia* group have been part of the diversification associated with breeding, and that they may play a role in modifying gene expression patterns in the flax genome, which can lead to diversified phenotypes.

## Results

### Comparison of TE copy number between flax accessions

**Fig. 1:**
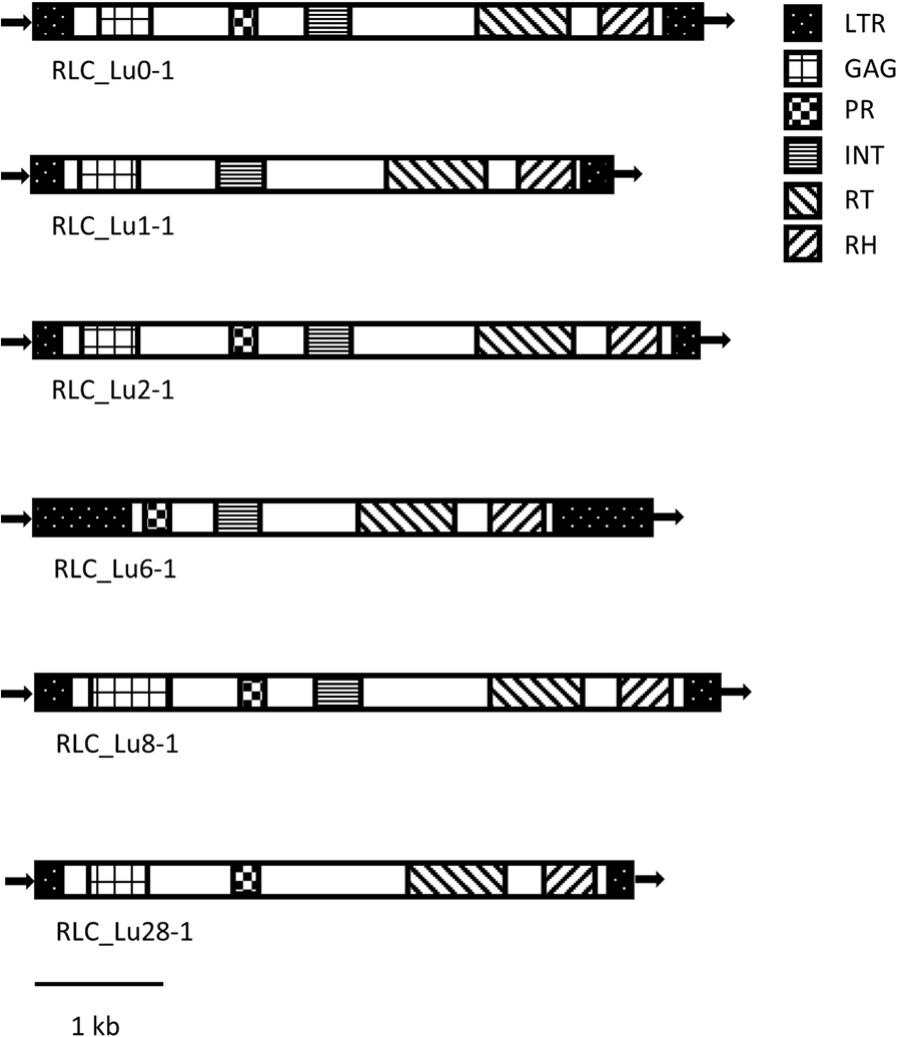
Diagrams of representative Ty1-*copia* TEs. Long terminal repeat (LTR), group-specific antigen domain (GAG), protease (PR), integrase (INT), reverse transcriptase (RT), RNase H (RH)

To compare the abundance of TE families between flax cultivars, we designed reverse transcriptase (RT) primers (Additional file 1A) from six selected Ty1*-copia* elements representative of six retrotransposon families, and used quantitative PCR (qPCR) to measure their abundance in 14 diverse flax accessions belonging to either oil or fiber, or spring and winter types (Table 1). The retrotransposon families were selected because previous analysis showed them to have LTRs with high similarity and conserved protein domains [17], suggesting the elements had been recently active, and may therefore be expected to be polymorphic between cultivars. Families were named according to previously suggested conventions (see Methods [45]) as: RLC_Lu0, RLC_Lu1, RLC_Lu2, RLC_Lu6, RLC_Lu8 and RLC_Lu28. From each family a representative sequence which showed conserved sites for primer design among family members was selected (see additional selection characteristics of representative sequences in the Methods section). Four of the six representative sequences from the selected retrotransposon families had 100% similarity in their LTRs and two had LTRs over 99% similar, indicating insertion of these elements in the last 200,000 years (Additional file 2). Similarly, we identified the five expected protein domains from Ty1-*copia* elements in half of the representative sequences, and four domains in the other half (Fig. 1).

**Table 1:** Cultivars used for transposon display

Cultivar	Type
Stormont Cirrus	fiber spring
Aurore	fiber spring
Belinka	fiber spring
Drakkar	fiber spring
Evea	fiber spring
Hermes	fiber spring
Violin	fiber winter
Adelie	fiber winter
rdf*	oil spring
Bethune	oil spring
Lutea	oil spring
Blizzard	oil winter
Oleane	oil winter
Oliver	oil winter

*rdf is a mutant derived from Bethune and therefore cannot be classified as a cultivar per-se. It should be referred as an accession

**Fig. 2:**
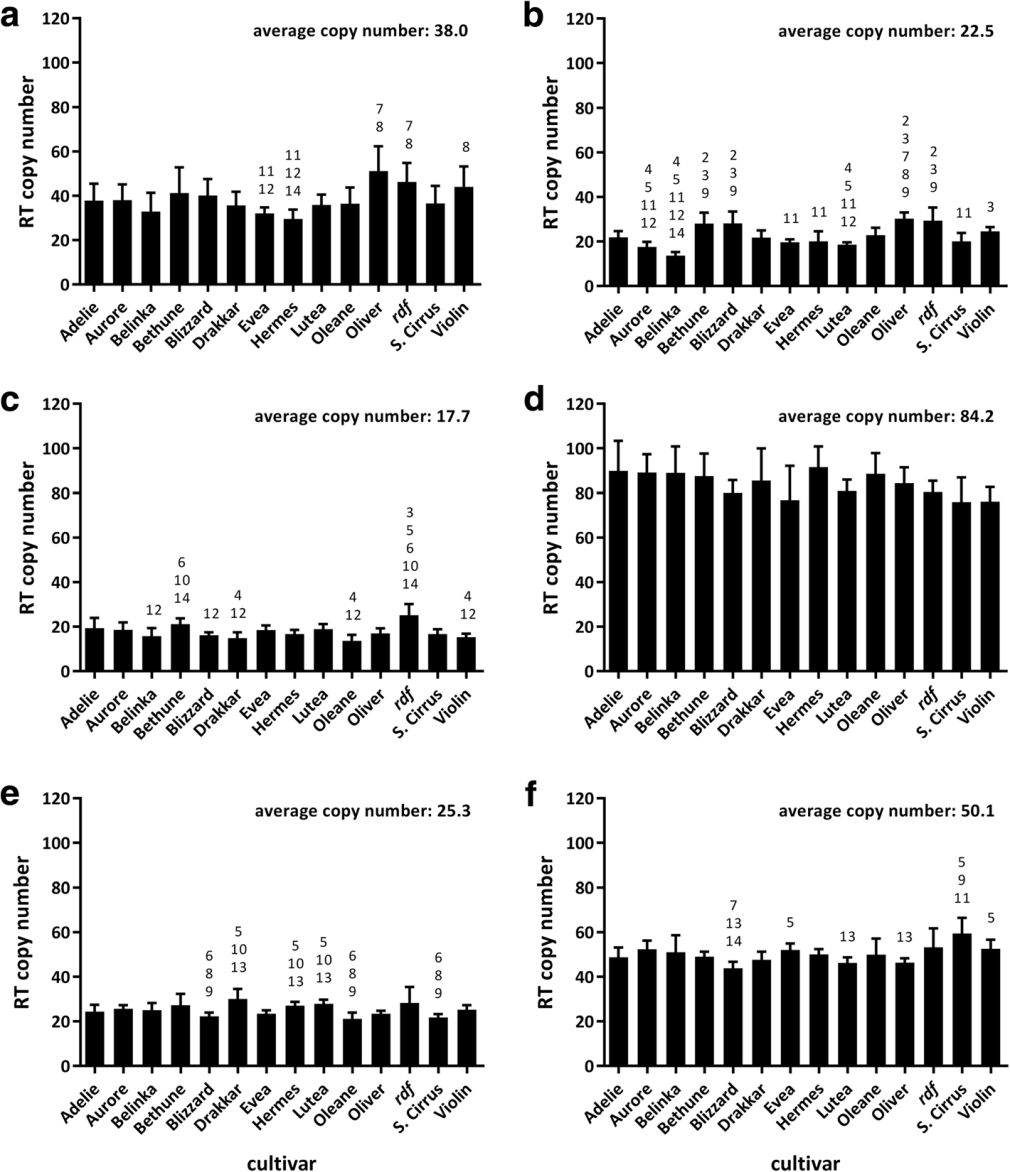
Absolute quantification of Ty1-*copia* retrotransposon families. The quantification was performed in 14 flax cultivars, based on amplification from their reverse transcriptase (RT) domains. The log10 of molecule copy number (mcn) was calculated using an online tool (see text) that accounts for plasmid+insert size. This value was used along Ct to generate standard curves to calculate molecule copy numbers for RTs, which were normalized to ETIF1 to find absolute copy number. Families depicted are: **a** RLC_Lu0, **b** RLC_Lu1, **c** RLC_Lu2, **d** RLC_Lu6, **e** RLC_Lu8, **f** RLC_Lu28. *Error bars* = standard deviation. Numbers above represent significant differences of the respective cultivar to other cultivars (Dunn’s multiple comparison test *p ≤* 0.05) which are numbered as: *1*. Adelie, *2*. Aurore, *3*. Belinka, *4*. Bethune, *5*. Blizzard, *6*. Drakkar, *7*. Evea, *8*. Hermes, *9*. Lutea, *10*. Oleane, *11*. Oliver, *12. rdf*, *13*. Stormont Cirrus. *14*. Violin. The average copy number for all cultivars in each TE family is also indicated

Quantitative PCR showed differences in TE family abundances (Fig. 2). When averaged across cultivars, family RLC_Lu2 presented the lowest copy number per haploid genome (17.7), with families RLC_Lu1, RLC_Lu8 and RLC_Lu0 following with 22.5, 25.3 and 38.0 copies respectively. Finally, family RLC_Lu28 had 50.1 copies per haploid genome, while family RLC_Lu6 had the largest average copy number of all with 84.2 copies. In order to validate these experimental findings, the copy number of each TE family was also estimated for the sequenced CDC (Crop Development Center) Bethune cultivar (herein named Bethune) by BLAST alignments of primer pairs used for quantitative PCR results. The expected copy number of TEs in each family estimated by BLAST alignments, was supported by the quantitative PCR results, showing a correlation of 0.85, and demonstrating the validity of the analysis. Non-parametric tests supported a significant variation in copy number for each family between cultivars in all cases (*p* ≤ 0.0027, Kruskal-Wallis test). Moreover, adjusted *p*-values (Dunn’s test) for all pairwise comparisons showed significant differences between some accessions for all TE families tested with the exception of family RLC_Lu6 (Fig. 2). No single cultivar or group of cultivars showed a clear expansion of all TE families, but the statistical analysis demonstrated a variability that could be exploited through the use of SSAPs.

### Identification of polymorphic TE insertions using SSAP

Having demonstrated significant variation in TE copy number between flax accessions, we used SSAP to identify individual insertions that were polymorphic between the accessions. We identified seven LTR primers that consistently amplified distinct bands from the same six Ty1-*copia* families used for copy number quantification (Additional file 1B). Two primers were used for family RLC_Lu1 because they generated distinct patterns and resulted in additional polymorphisms. The seven primers were used on to amplify DNA from each of the 14 flax accessions (Table 1), to generate the SSAP profiles (an example is shown in Additional file 3). A total of 219 bands were scored, from which 140 were polymorphic (63.9% - Table 2). The primers with the lowest number of average bands were LTR-RLC_Lu1-primer1 and LTR_RCL_Lu1-primer2 (8.1 and 8.5 respectively); however, they also showed the highest rate of polymorphism (96.6 and 90% respectively). Conversely, LTR-RLC_Lu6-primer3 produced the highest number of bands across cultivars, with an average of 49.6, but showed the lowest rate of polymorphism (25%). The number of expected TEs from the Bethune genomic sequence analysis (Additional file 1A) was also correlated (*r* = 0.80) with the number of scored bands in the same cultivar (Table 2), showing consistency between methods.

**Table 2:** SSAP band scoring and polymorphic bands

Accession	LTR-RLC_Lu0-primer-3	LTR-RLC_Lu1-primer-1	LTR-RLC_Lu1-primer-2	LTR-RLC_Lu2-primer-1	LTR-RLC_Lu6-primer-3	LTR-RLC_Lu8-primer-1	LTR-RLC_Lu28-primer-1	Totals
*rdf*	18	12	13	12	50	8	20	133
Bethune	18	10	14	12	50	8	20	132
Lutea	14	4	6	12	48	8	20	112
Oleane	14	4	5	9	48	8	22	110
Blizzard	16	10	9	9	49	10	17	120
Oliver	17	10	10	11	49	8	20	125
Violin	16	12	11	12	49	10	24	134
Adelie	14	7	8	14	50	10	20	123
S. Cirrus	13	8	6	17	48	5	23	120
Evea	14	10	9	13	52	8	23	129
Drakkar	15	9	8	14	50	12	23	131
Hermes	14	8	9	13	50	8	24	126
Belinka	20	4	5	13	50	8	22	122
Aurore	18	6	6	11	51	9	23	124
Average number of bands	15.8	8.1	8.5	12.3	49.6	8.6	21.5	17.8
Scored positions	28	29	30	22	56	22	32	219
Polymorphic positions	17	28	27	16	14	19	19	140
%Polymorphism	60.7	96.6	90.0	72.7	25.0	86.4	59.4	63.9

**Fig. 3:**
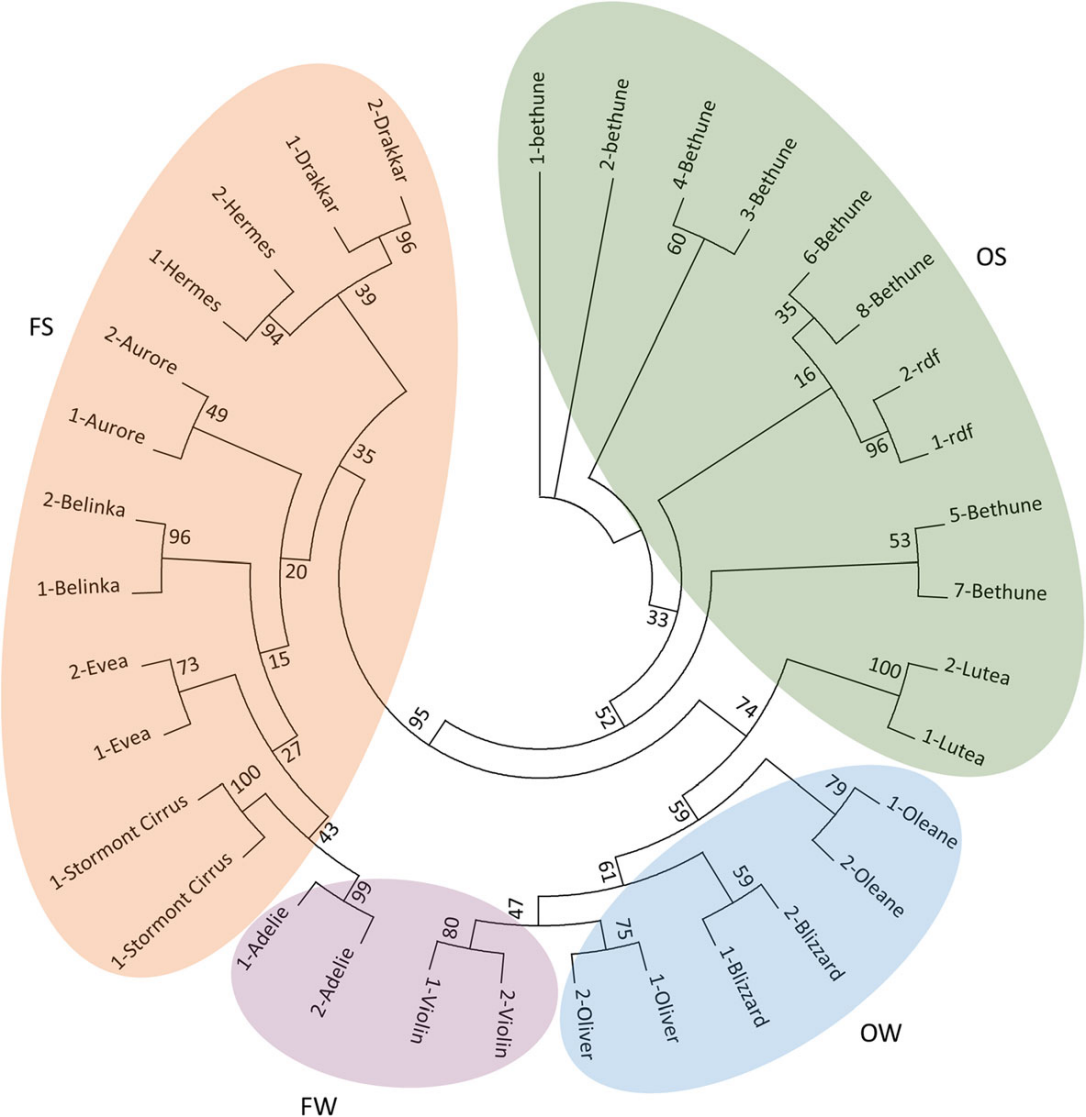
ML tree using 14 flax cultivars. The ML tree was built using a general time reversible model for binary data and 1000 bootstrap replicates (bootstrap support is shown for each branch). The colored groupings reflect different flax types: *orange* (fiber spring - FS), *purple* (fiber winter - FW), *green* (oil spring - OS), *blue* (oil winter - OW). Two biological replicates were used per cultivar with the exception of Bethune with eight replicates

SSAP bands were converted into a binary matrix (band presence = 1, absence = 0), which was used to construct a maximum likelihood (ML) tree using IQ-TREE v1.4.4 [46]. For the most part, oil (linseed)-types were more similar to each other than they were to fiber-types, with the exception of the winter fiber variety, Violin (Fig. 3). A grouping pattern was also discerned for the dichotomy between spring and winter types, with the exception of Adelie, a winter fiber type which seemed closer to spring fiber types, and Lutea, a spring oil type which was closer to the winter oil types (Fig. 3).

No single band could distinguish all linseed-types from all fiber-types, or all winter-types from spring-types, however, the definition of a cultivar as either a winter-type or spring-type can sometimes be ambiguous depending on the breeding program from which it originated [40]. Within the linseed-types, Bethune and *rdf* (reduced fiber) had the most SSAP sequenced bands in common, followed by Oliver and Blizzard (Additional file 4). Bethune and *rdf* had 4 bands which were only present in those two accessions: band 25 (LTR-RLC_Lu1-primer1), band 15 (LTR-RLC_Lu1-primer2), band 5 (LTR-RLC_Lu2-primer1), and band 4 (LTR-RLC_Lu8-primer1). At the same time, Oliver and Blizzard had 2 bands that were exclusively in those two cultivars: band 17 (LTR-RLC_Lu0-primer3), and band 14 (LTR-RLC_Lu1-primer1).

In the case of the fiber-types, Evea, Drakkar, and Hermes shared the most bands. A band derived from LTR-RLC_Lu2-primer1 (band 10) was present in seven of the eight fiber-types tested and was absent from the linseed types (Additional file 4); the same was true for band 6 from LTR-RLC_Lu28-primer1 but the band was also present in *rdf* and Bethune (linseed types). One more band from LTR-RLC_Lu28-primer1 was common to eight fiber-types (band 12), although in this case, one linseed-type (Lutea) also had the band. One single band was present only in all spring-fiber types (LTR-RLC_Lu2-primer1, band 4), but this one was also present in Lutea (Additional file 4).

### Analysis of flax genomic sequences targeted by polymorphic insertions

From the 140 polymorphic bands detected using SSAPs, 99 were successfully excised and sequenced. Most of the failed sequencing occurred with the highest molecular weight bands, which were either difficult to re-amplify or did not otherwise produce high quality sequence. Some of the resulting sequences were redundant, probably because the restriction enzyme used for the SSAPs found cryptic sites in larger fragments; alternatively, different cultivars could have the same TE insertion accumulating mutations that generate a new restriction site, resulting in bands with different electrophoretic mobility, but which represented the same insertion event. After filtering for residual redundancies, sequences where the restriction site fell inside or just besides a TE, and sequences where no LTR could be identified, 66 unique insertion sites where found (Additional files 4 and 5). Each insertion was classified according to its Ty1-*copia* family and was mapped to the genome assembly according to the annotation deposited in phytozome (Additional file 4). Of the 66 insertions, 14 (21.2%) interrupted annotated exons, 30 (45.5%) were in introns, 11 (16.7%) were within 1 kb of a gene opening reading frame (upstream or downstream), and 11 (16.7%) were characterized as intergenic (where the TE was inserted at a distance of more than 1 kb from any annotated gene). Altogether, more than 83.3% of the cloned TE insertions mapped within genes, or within 1 kb of a gene. For insertions in introns, exons, or within 1 kb of the CDS, the inferred transcription sense strand of the TE and gene were the same in 30 cases. Conversely, in 28 cases, the TE and associated gene were transcribed from opposite strands (Additional file 4).

To validate the results of the SSAPs, we conducted genomic PCR assays of 28 selected insertions (Additional file 6) on each of the 14 flax accessions. We designed from each TE insertion site sequence (Additional file 5) one specific primer complementary to the flanking genomic DNA (listed in Additional file 1C) and performed PCR with the corresponding LTR-RLC SSAP primer (Additional file 1B). Nineteen (67.9%) of the validated insertions showed a perfect match or nearly perfect match to the polymorphisms initially assessed with SSAPs, while the remaining bands had different levels of disagreement (Additional file 6).

We used Gene Ontology (GO) categories to classify the genes that were found to be associated with polymorphic TE insertions. The genes represented 15 cellular components, 12 molecular functions, and 14 biological processes (Additional file 7). Nine genes where classified as responsive to stress, four in DNA or RNA metabolism, nine in cell organization and biogenesis, nine corresponded to protein metabolism, five were related to transcription, three to transport, five to development, six involved in signal transduction and one was related to electron transport or energy processes. None of the categories were enriched when the Arabidopsis orthologs were compared to the background of all annotated genes using AgriGO (data not shown).

### qRT-PCR analysis of selected genes with polymorphic TE insertions

**Fig. 4:**
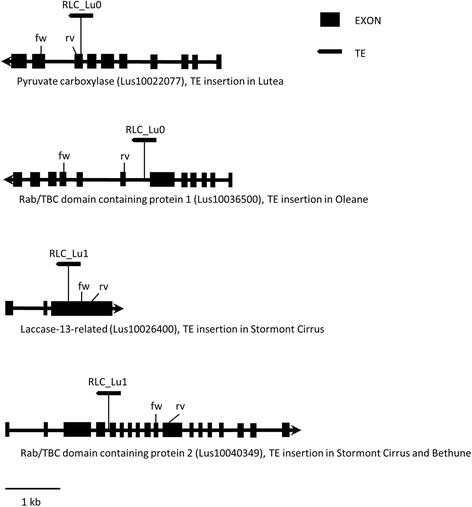
Diagrams of genes bearing Ty1-*copia* TE insertions. The location of the primers used to test for changes in gene expression is displayed. Gene expression was tested in five flax cultivars (Lutea, Oleane, S. Cirrus, Bethune and Oliver), which were polymorphic for the insertions. The name of the TE family is above each represented TE. Orientation of the genes and TEs is as depicted after mapping using phytozome. Genes are drawn according to scale while TEs (not to scale) are depicted only to show insertion positions

**Fig. 5:**
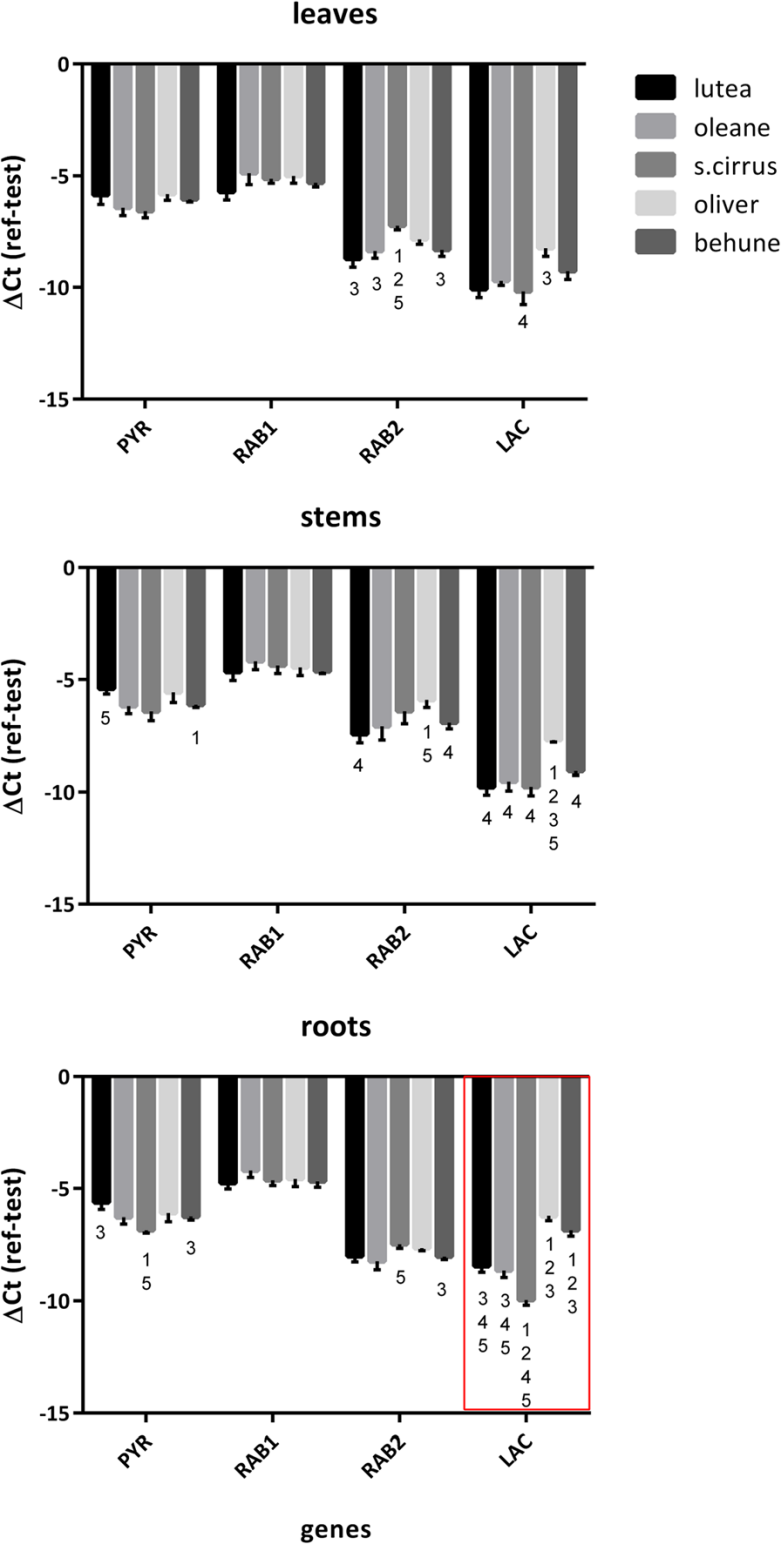
Normalized gene expression of four genes bearing TE insertions in three different tissues. Each ΔCt corresponds to the average of four biological replicates, each with three qRT-PCR technical replicates. PYR (Pyruvate carboxylase – Lus10022077), RAB1 (Rabgap/TBC domain containing protein 1 – Lus10036500), RAB2 (Rabgap/TBC domain containing protein 2 – Lus10040349), LAC (Laccase-13-related – Lus10026400). All pairwise comparison in each gene and tissue, were performed using unpaired two-tailed *t-*tests, and significant differences were calculated after Bonferroni correction (*p* < 0.005). Numbers above represent significant differences of the respective cultivar to other cultivars which are numbered as: *1*. Lutea, *2*. Oleane, *3*. Stormont Cirrus, *4*. Oliver, *5*. Bethune. *Error bars* = standard deviation. The *red outline* in roots depicts the only expression pattern in agreement with the insertion of a TE (TE inserted in the Laccase gene of S. Cirrus and absent in the rest of the cultivars)

To assess effects of TE insertions on gene expression, we selected four flax genes from Additional file 4 that were expected to be constitutively expressed under normal conditions, so that any effects of TE insertion on gene expression could be detected. In making this selection, we relied partly on flax RNA-seq data of control plants from an experiment on the flax-fusarium interaction performed in our lab (Galindo-González & Deyholos, in preparation), and on comparisons to the presumptive Arabidopsis orthologs of our flax genes, since extensive transcript expression data is available for Arabidopsis (ThaleMine - [47, 48]). Four flax genes were selected for qRT-PCR analysis: Pyruvate carboxylase (Lus10022077), a Laccase-13-related gene (Lus10026400), and two Rabgap/TBC domain containing proteins (Lus10036500 and Lus10040349). The two Rabgap/TBC domain containing proteins had 83.6% nucleotide identity, and bore the TE insertions in different regions (Additional file 4). Additionally, in order to study potential positional effects of the TE insertions, we selected genes harbouring TEs in exons or introns and TE-gene associations in sense or antisense orientation (Fig. 4). Primers were designed downstream the insertion following the theoretical gene transcription orientation (Fig. 4). Five flax accessions that were polymorphic for insertions in these four genes were selected to be assayed on three different tissues (leaves, root and stem) by qRT-PCR: Lutea (TE insertion in exon 8 of pyruvate carboxylase), Oleane (insertion in intron 6 of first Rabgap/TBC domain containing protein); Stormont Cirrus (TE insertion in exon 3 of the Laccase-13-related gene), and an insertion in a second Rabgap/TBC domain containing protein of intron 4, which was also present in Bethune (Fig. 4); and Oliver, which had no TE insertions in any these four genes. The results of the qRT-PCR analysis showed several differences (some with statistical significance) in the relative expression of the genes tested (Fig. 5). Only the laccase gene harbouring a TE insertion (Stormont Cirrus accession) displayed a significant decrease in root gene expression (*p* < 0.005) compared to homologous laccase gene of the other four cultivars evaluated that did not bear the insertion (Fig. 5). There was also a significant difference between the expression of Lutea/Oleane when compared with Oliver/Bethune, but the expression in Stormont Cirrus was lower than in any other of the four cultivars. The three other genes did not show decrease in gene expression in agreement with the accession containing the TE insertion.

## Discussion

### TE activity and genomic copy number

Quantitative PCR using six Ty1*-copia* families in 14 flax cultivars demonstrated copy number variation between TE families, but also within each family between cultivars. Artificial selection through plant breeding involves subjecting plants to diverse stress conditions (e.g. drought, cold), and growing them under agricultural and laboratory practices which are not always common in natural environments. Mobilization of TEs has accompanied the processes of breeding, as evidenced by the level of inter-varietal polymorphisms found using different transposon-derived markers [10, 12, 13, 42, 49].

A first clue that genomes diverge with respect to transposon history is a difference in abundance of specific TEs. Our approach to assess copy numbers of the selected TE families in flax followed a previous report that quantified the highly abundant BARE-1 retrotransposons from barley in several cultivars [50]. We found significant differences in copy numbers between cultivars for each TE family examined and differences when testing for multiple comparisons in five out of six TE families (Fig. 2). One of the most extreme examples of genome diversification due to active TEs in a plant genomes was demonstrated by amplifications of the *mPing* MITE (Miniature Inverted-repeat TE) in different rice landraces, where the element was actively transposing and ranged from 50 to more than 1000 copies [51]. While the differences in abundance of the Ty1*-copia* families we tested in flax were not as large as reported for *mPing* in rice, they were nevertheless, indicative of recent activity of these TEs.

In general, the absolute copy numbers we reported (Fig. 2), probably underestimate the actual abundance of the Ty1-*copia* families, due to frequent recombination and high mutation rates expected among LTR elements [52]. This results in modification of binding sites for qPCR primers or loss of internal retrotransposon domains, with concomitant creation of solo LTRs [53–55], which would not be accounted for by our method, based on amplification of the internal RT gene. Furthermore, the expected number of annealing sites from our BLAST analysis (Additional file 1A) is almost always lower than the calculated copy number by qPCR (Fig. 2) in Bethune (this happens for 5 out of 6 families). This is probably a result of unassembled genome regions which are yet to be reported in the database (in general regions which are difficult to assemble are rich in repeats such as TEs). Nonetheless, the copy numbers we reported for each family for Bethune (Fig. 2), were positively correlated with the number of TEs identified by BLAST alignment of primer binding sites to the Bethune genome assembly (*r* = 0.85), showing the validity of our approach. There was also a proportional high correlation between the TEs counted by BLAST alignment and the number of SSAP bands found for Bethune (*r* = 0.80). In this case the number of SSAP bands for Bethune (Table 2), was generally lower than the number of expected hits (Additional file 1A), and calculated TE copy numbers (Fig. 2) in each family, because this transposon display technique is only efficient for insertions located close to a restrictions site, and thus only reveals a subset of all insertions.

The highest estimated copy numbers and SSAP bands across all cultivars were found on family RLC_Lu6 (Compare Fig. 2 and Table 2). Interestingly, this family also had the lowest proportion of polymorphic bands (Table 2), and most of its flanking sequences were not related to gene regions (Additional file 4). An explanation for the insertion pattern and abundance of family RLC_Lu6 could be related to a lower level of negative selection, since TEs inserted in regions of low gene abundance, may not be as detrimental for the genome. Likewise, a lower level of polymorphism reflects inactivity, and it is therefore likely that family RLC_Lu6 expanded in the flax genome before breeding of these cultivars, and has been mostly quiescent since. In fact, this family’s representative sequence has the largest LTR divergence (Additional file 2), supporting this hypothesis.

Opposite to what happened with family RLC_Lu6, there are compelling clues that the lower copy number families from our study were active in the recent past as demonstrated by the differences in TE copy numbers between cultivars (Fig. 2), level of SSAP polymorphism (Table 2), their LTR similarity (Additional file 2), and that they relate more closely to genes (Additional file 4). This is in agreement with low copy number TEs catalogued as being inserted closer to genes [56], and also being more active in recent past than high-copy number TEs in plants like maize [57, 58]. Analysis of the maize genome suggest that the transition from low copy to high copy number TEs should be placed in the 10–100 copies range [56], which is in agreement with the difference between RLC_Lu6 and our low copy number families.

### SSAP markers associate with flax types

SSAPs were performed with the same six families for which we measured TE copy number. Based on 140 polymorphic bands, we produced a ML tree, in which accessions showed associations reflecting the division between fiber and oil types, with the exception of Violin, a fiber winter type that was closer to the oil winter types that to its other fiber winter partner (Adelie) (Fig. 3). Violin is a cultivar that behaves in the field like a dual purpose (oil/fiber) winter flax, and is genetically closer to an oil type, while Adelie has characteristics of a spring fiber (Jean-Paul Trouvé - personal communication). This would explain the close relationship of Violin to winter linseed types in the ML tree, as well as the close relationship of Adelie to the fiber spring types. Previously, the division of fiber and oil types was supported by molecular studies looking at genes closely linked to the distinct phenotypes of these groups. The *sad2* gene, involved in fatty acid metabolism, was used to determine an ancestral state of domestication of oil over fiber flax [59]. Additional candidate genes related to fiber development or oil metabolism that can distinguish between fiber and linseed varieties, were also found in a genetic diversity study by Soto-Cerda and collaborators [60]. The TE markers found in our study are therefore a good source of molecular variation that can be linked to genes involved in the divergence of flax types (see more below).

### Analysis of TE insertions and potential impact on genes

Characterization of insertion sites from polymorphic bands of the SSAPs in flax cultivars, evidenced a high percentage of association of TEs to genes. Polymorphic TE insertions can result in genome divergence through genome restructuring, gene mutation and regulation changes (e.g. LTRs upstream of genes can change expression patterns), which at the same time depend on the TE’s insertion site preference, and regulation of their transposition by host mechanisms (e.g. epigenetic control). While the mechanisms for insertion site selection are still not completely understood, insertional bias is evident for certain TE families. Young *Copia*-like retrotransposons have been shown to insert more randomly than *Gypsy*-like elements in Arabidopsis, and are associated with euchromatic gene-rich regions [26, 27]. Similarly, we previously found that in flax, recently inserted Ty1-*copia* elements were non-randomly associated with gene regions and constituted the largest superfamily of TEs in the flax genome [17]. Our results here confirmed that numerous Ty1-*copia* TEs are biased towards insertion close to or inside genes. GO (Gene Ontology) classification (Additional file 7), however, showed no bias towards specific functional categories of genes.

TD has been often used to find polymorphic markers to study intraspecific genetic diversity [12, 13, 42, 61, 62], but these types of studies rarely characterize polymorphic insertion sites with detail at the sequence level. We successfully sequenced 66 non-redundant insertions in different genomic locations. Analysis of these insertion sites showed some interesting genes that could be related to agronomic traits, and represent potential candidates for future studies. For example, band 11 from LTR-RLC_Lu0-primer3 (Additional file 4), was characterized as a TE insertion on intron 19 of Pyruvate dehydrogenase E1. This gene is involved in fatty acid biosynthesis, and has been identified as potentially associated with divergent selection between flax types [60]. We also found a TE insertion on exon 2 of a Pinoresinol-lariciresinol reductase 3 gene (PLR 3) for cultivars Bethune and the associated mutant accession *rdf* (LTR-RLC_Lu2-primer1, band 5 - Additional file 4). PLR 1 is a key enzyme in flax lignan biosynthesis [63, 64]; lignans act as antioxidants, as well as having beneficial effects on human health [65]. Another interesting gene annotated as a Laccase-13-related, had a TE insertion on exon 3 (LTR-RLC_Lu1-primer1, band 18 - Additional file 4) that was present in six fiber cultivars. Laccases catalyze the last step in lignin biosynthesis [66], and downregulation of lignin biosynthetic genes (including laccases) has been associated with the hypolignification of bast fibers, a desirable characteristic for easier harvesting [67, 68]. Another gene with a TE insertion, was 1-aminocyclopropane-carboxylate synthase 2-related (LTR-RLC_Lu8-primer1, band 7 - Additional file 4), which was previously identified as Lu-ACS5 (1-aminocyclopropane-carboxylate synthase 5) [69]. ACS enzymes are involved in ethylene synthesis, and a previous study of ACS gene expression in flax roots showed that transcript abundance of ACS5 did not change in response to treatment with auxin antagonists, although transcripts of four other ACS genes did. Whether or not this might be related to the TE insertion is yet to be investigated. We also found a TE overlapping with a WRKY27 transcription factor (LTR-RLC_8-primer1, band 11 - Additional file 4). Mutants of this gene in Arabidopsis have delayed wilting upon infection with the bacterial pathogen *Ralstonia solanacearum*, showing that the gene might be a negative regulator of defense response [70].

Our results showed that 83.3% of insertions disrupt exons, or introns or are otherwise in close proximity to coding regions (Additional file 4). Fourteen (21.2%) of the characterized insertions in our study disrupted exons. While the most common result of an exon disruption is loss of gene function, this loss can result in a desirable agronomic trait. As an example, glutinous rice is the product of a retrotransposon disrupting an exon of the granule-bound starch synthase gene [71]. We also found 30 TEs mapped to introns. Intron insertions can result in different patterns: An LTR-retrotransposon insertion in different introns of a MADS-box transcription factor of different apple varieties causes transcript suppression leading to seedless fruits [72], and waxy kernel phenotypes in maize result from alternative splicing patterns caused by retrotransposon insertions in introns of an amylose biosynthesis gene [73]. Regulation of expression can also result from TEs that do not disrupt the coding sequence; we found 10 insertions within 1 kb of genes. Examples of the impact of extragenic insertions include: insertions of LTR retrotransposons adjacent to MYB genes involved in anthocyanin biosynthesis resulting in skin color variation in grape cultivars [74], and in the production of blood oranges [28]; insertion of a retrotransposon in the 5′ UTR region of a vernalization gene (VRN1), which allows winter wheat to grow as a spring-type wheat [75]; and an increase in disease resistance to rice blast due to an insertion of an LTR retrotransposon in the promoter of the *Pit* resistance gene [29].

We found a particular example of a TE carrying an F-box domain protein between its two LTRs. This TE (band 8 from LTR-RLC_Lu6-primer3 in Additional file 4) has a recognizable RNAse H (ribonuclease H) domain near the 3′ LTR and therefore represents a functional retrotransposon that has acquired a gene. This gene capture and capacity to mobilize the gene is known as transduplication, and has been widely seen with over 3000 Pack-MULEs (Mu-like Elements) in rice that have captured over 1000 genes [76]. Retrotransposons in rice and sorghum have also been shown to capture numerous genes [77].

### TE impact on flax gene expression

A preliminary assay was performed to test the effects of flax TEs on gene expression, using qRT-PCR on four genes with insertions in either exons or introns. Only the Laccase gene demonstrated a significant decreased transcript abundance in roots that correlated to the presence of the TE insertion: the cultivar Stormont Cirrus harbouring a TE insertion had lower relative transcript abundance than the other cultivars without the insertion (Fig. 5). Observation of the qRT-PCR bands on a gel (not shown), demonstrated that the expected band was present in all cultivars, which would mean that likely scenarios for repression would be: i) anti-sense gene transcripts generated from readout of the TE inserted in opposite orientation of the gene (Fig. 4), that could potentially be used for the generation of small RNAs tagging the gene for inactivation via methylation [34, 78], or, ii) the generation of a different splice form, which conserves the exon tested by qRT-PCR, but has reduced transcript abundance as a consequence of the modification [71, 73]. Other statistical differences where found, but none of these were in agreement with the presence or absence of a TE insertion. We believe these differences could be related to cultivar specific differential expression (Fig. 5).

For the Pyruvate carboxylase gene, we expected that the TE insertion in exon 8 would result in transcript alterations for Lutea but this was not the case. For both of the Rabgap/TBC domain genes which had insertions on introns, no impact on gene expression was evidenced.

These results show no common mechanisms by which these insertions may alter gene expression. Insertion on exons would be expected to be directly disruptive but only in one of two cases a change was noticed. Opposite orientation of TE-derived transcripts could create epigenetic-mediated gene silencing [34, 78], but this should depend on actual transcriptional activation of our TEs which might not be happening under our conditions. And TEs inserted in introns could change gene expression or splice forms, but this does not always happen, and alternate transcripts can be created from one single gene with an insertion [73]. Finally, if the TE insertion is present in just one allele of the gene, the TE effects can be masked by the other allele functioning normally.

In future studies, stress conditions or treatments which upregulate genes with inserted TEs might proof to be a better strategy to discern if gene expression levels are affected by the TE, for two reasons: i) the stress can generate a higher response of the host gene that can be more distinct that a low constitutive expression if the TE really alters gene expression, and ii) the stress may also upregulate the transcription of the TE, increasing the chance of readout transcripts that can be used for the production of small RNAs that can mediate silencing. These examinations should be coupled with experiments: i) to assess the production of small RNAs and methylation state of the gene, and ii) revise if TE insertions are homozygous and if different splice forms are produced from the host gene.

## Conclusions

Based on our findings, we can conclude that there have been recent active retrotransposition events since breeding started for the tested cultivars. The TE markers found using SSAPs were useful to separate the major flax types, and their level of polymorphism further showed that they have an impact on diversifying flax cultivars. While not all flax TEs examined fall in gene-rich regions [17], we now know that the transposition of most studied families here is biased towards these regions and their study constitutes a good source of novel mutations that can be used to find potential linkage to diversifying phenotypes, which is the basis for creating new cultivars. In fact, strong proof of TE-mediated diversification exists in closely related species of Arabidopsis [35] and rice [52] and in cultivars of rice [51] and maize [79]. No matter what the adaptive fate of these insertions may be, the mobilization of TEs among flax cultivars constitutes a powerful tool in diversity studies. However, understanding how these insertions influence genome restructuring and shape gene evolution requires studying related cultivars and species to determine what insertions may be under purifying selection and which ones are being positively selected as part of the normal functioning of the genome. The TE insertions found here, in different gene regions and in different orientations, open the door to study their potential influence on gene regulation on a case by case basis.

## Methods

### Plant material

For determination of TE family copy numbers, eight plants from each of 14 flax cultivars or accessions (Table 1) were grown in a growth chamber at the University of Alberta under the following settings: seeds sown in pods with a 50/50 soil/sand mix, 16 h of light/8 h of dark (0.132 μMoles of light), 22 °C, 50% humidity. Aerial sections (stems and leaves) were harvested after 2 weeks of growth and instantly frozen with liquid nitrogen in 2 mL tubes. These cultivars were selected based on their expected broad genetic base (this fact is more common for spring types). Only two representatives of fiber-winter were selected due to availability, because these cultivars are uncommon and not widely grown or bred.

For SSAPs, 14 flax cultivars were used (Table 1). Plants were grown in greenhouse conditions (14 h of light, 24 °C day/20 °C night, 40% humidity) at the National Institute for Agronomic Research (INRA) in Versailles, France. Seeds were sown in pods with a 50/50 soil/sand mix, and left to grow for 2 weeks before aerial sections were collected in 2 mL tubes and instantly frozen in liquid nitrogen.

For testing the expression of genes bearing polymorphic TE insertions among cultivars additional plants of each cultivar were grown in the same growth chamber at the University of Alberta under the same conditions used for determination of TE copy number. Stems, 5–10 young leaves (including the apical meristem) and roots were harvested after 2–3 weeks of growth.

### Nucleic acids extraction and cDNA synthesis

The samples for SSAPs were ground with a plastic pestle maintaining the tube in liquid nitrogen until achieving a fine powder. Samples for SSAP validation, transposon families copy number determination and gene expression were ground adding an autoclaved 5.6 mm stainless steel bead, and using a Retsch MM301 mixer mill (Retsch, Haan, Germany) with two cycles of 1 min at 20 Hz.

DNA extraction was performed using the DNeasy Plant Mini Kit (QIAGEN, Venlo, The Netherlands). Sample quantification was performed with a Nanodrop ND-1000 spectrophotometer (Thermo Fisher Scientific, Waltham, MA, USA).

RNA was extracted using the RNeasy Plant Mini Kit (QIAGEN, Venlo, The Netherlands), and quantity was assessed using a Nanodrop ND-1000 spectrophotometer (Thermo Scientific, Waltham, MA, USA). A DNAse treatment was performed for 30 min at 37 °C after extraction with DNase I (Thermo Scientific, Waltham, MA, USA). For cDNA synthesis 500 ng of DNAse treated RNA were used to perform reverse transcription using the RevertAid H Minus Reverse transcriptase under the manufacturer specifications and using oligo dT (18) (Thermo Scientific, Waltham, MA, USA). To test for residual contamination of DNA a PCR was performed with primers from the eukaryotic translation initiation factor 3E (ETIF3E) which has constitutive expression in the tested tissues (Additional file 1E). The PCR was run with 1× buffer, 2 mM MgCl_2_, 0.2 mM dNTPs, 0.4 μM of each primer, 5 ng of cDNA and 1.5 units of Taq polymerase (Thermo Scientific, Waltham, MA, USA). Cycling conditions were 94 °C for 2 min, followed by 35 cycles of 94 °C for 30 s, 60 °C for 30 s and 72 °C for 1 min, finalizing with an extension at 72 °C for 5 min.

### TE primers

Retrotransposon sequences were obtained from our previous study on transposable elements of flax [17]. To design Ty1-*copia* primers, TE families were first defined: family membership of a Ty1-*copia* element was established with a threshold similarity of at least 80% in at least 80% of the aligned sequence, following previously established rules for family membership [45]. The comparison was performed on the 554 non-redundant reverse transcriptase (RT) domain sequences, which were first predicted using RepeatExplorer [80, 81], and then used as input for CD-HIT-est [82, 83] using an identity cutoff and minimal alignment coverages of 0.8. Families were named using the suggested designation of class, order and superfamily [45], followed by a species designation, and a number corresponding to the specific TE (e.g. Retrotransposon-LTR-Copia from *Linum usitatissimum* family 0, representative sequence 1 = RLC_Lu0-1). Selected families with evidence of recent insertion (high similarity among its LTRs and conserved domain proteins) were selected for primer design. To calculate the insertion age of the TEs, first LTR pairs from each element where aligned using ClustalW from MEGA v6.06 [84, 85] and the Kimura two-parameter method [86] was used to calculate nucleotide substitution. Then, the age of insertion was estimated as *t = K/2r*, where *K* corresponds to the nucleotide substitution per site and *r* corresponds to the nucleotide substitution rate which in this case was taken from a previous study used for dating LTR retrotransposons in Arabidopsis [26]. Presence of the main protein domains in Ty1-*copia* elements: GAG (group-specific antigen), PR (protease), INT (integrase), RT (reverse transcriptase) and RH (Ribonuclease H), was assessed using conserved domains from NCBI [87, 88], and RepeatExplorer [80, 81].

To design reverse transcriptase (RT) primers to assess TE copy number (see below), the RT nucleotide sequences from all members in each TE family were aligned using ClustalW [84] from MEGA v6.06 [85] using the following parameters: a gap opening penalty of 15 and a gap extension penalty of 6.66 for both pairwise and multiple alignments, DNA weight matrix – IUB, transition weight of 0.5, negative matrix off and delay divergent sequences that have less than 40% similarity. For each family, one representative sequence bearing conserved sites for primer design from all (or most) family members, was used as input for Primer3 [89, 90] with the following parameters: primer size range between 18 and 24 bp, temperature between 57 and 63°, product size 100–200 bp, and GC content between 40 and 60% (the rest of the parameters were left by default). Candidate representative sequences from each alignment were chosen based on preliminary bioinformatics analysis showing protein domain conservation (at least 4 out of 5 of expected domains (GAG, PR, INT, RT and RH), and high LTR similarity to all members of the family (not shown). The reference gene used to normalize copy number was ETIF1 (eukaryotic translation initiation factor 1) which has been previously tested in flax quantitative gene expression [91]. Selected RT primer pairs (Additional file 1A), were aligned to the flax genome (Bethune) using BLAST to get an estimate of the expected copy numbers per TE family. From two primer pairs designed per family (six families in total) for qPCR (see below), the one with a better standard curve was selected in each family. The expected number of reported hits to the flax genome (Additional file 1A) reflects matches where both primers from the pair have 100% identity and 100% coverage to the match site, with no gaps. Additionally, for all matches reported in all families, the distance between the primer pair reflects the range of the expected size displayed in Additional file 1A.

To design primers for SSAPs, LTRs from all family members in each of the selected families were aligned following the same alignment parameters as for the RT sequences when designing primers to assess TE family copy number. After filtering largely divergent and redundant sequences from the alignment, the complete retrotransposon sequences were checked for the presence of internal *Eco*RI sites in order to minimize the chance of amplifying retrotransposon internal regions. The LTR alignments were then scanned for a region that is conserved among most aligned elements of the family, to maximize the generation SSAP bands. A representative sequence was selected in each family (Additional file 2), and the conserved region was then used as input for Primer3 [89, 90] along with the primer corresponding to the *Eco*RI adapter with the following parameters: primer size range between 20 and 24 bp, temperature between 55 and 62° and GC content between 30 and 70% (the rest of the parameters were left by default). A total of 19 primers were designed for six TE families, but only seven were used for the final experiment (Additional file 1B).

### Transposon family copy number

To find the absolute copy number of TEs from each of the families used in the SSAPs (see below), we performed qPCR on DNA samples from the 14 cultivars using reverse transcriptase (RT) TE primers (Additional file 1A). The amplifications were then compared to standard curve dilutions of the cloned amplified RT fragments (see below).

PCR to amplify the RT regions to be cloned was performed with 1× Taq buffer, 2 mM of MgCl_2_, 0.2 mM dNTPs, 0.2 μM of each primer and 1 unit of recombinant Taq polymerase (Thermo Fisher Scientific, Waltham, MA, USA). Cycling conditions were as following: 94 °C for 2 min followed by 35 cycles of 94 °C for 30 s, 60 °C for 30 s and 72 °C for 1 min, with a final extension at 72 °C for 5 min. PCR was run on a 1% agarose gel at 90 V for 60 min and the expected amplicon size was assessed. Then, the bands were eluted using the Wizard SV gel and PCR clean-up system (Promega, Madison, WI, USA). Eluted products were quantified using a Nanodrop ND-1000 spectrophotometer (Thermo Fisher Scientific, Waltham, MA, USA). For sequencing, 150 ng of the eluted product was used along with a primer at a final concentration of 0.25 μM (forward or reverse primers corresponding to the same primers used for PCR). Sequencing reactions were performed with the BigDye terminator v3.1 cycle sequencing kit (Applied Biosystems - Thermo Fisher Scientific, Waltham, MA, USA) using a 3730 Genetic Analyzer equipment (Applied Biosystems -Thermo Fisher Scientific, Waltham, MA, USA). Sequencing products were aligned with the original RT sequences to confirm that amplification products were as expected.

To clone the amplification products, ~5–8 ng of the insert (this varied depending on the amplicon size) were cloned into the PGEM-T vector II system (Promega, Madison, WI, USA) to create a 3:1 (insert:vector) molar ratio. Ligation products were transformed into JM109 high-efficiency competent cells (Promega, Madison, WI, USA), following the manufacturer recommendations. One hundred microliters of the transformed cultures were plated into LB-agar plates with 2% X-gal, 20% IPTG and 50 ng/μL of ampicillin. Cultures were incubated overnight (ON) at 37 °C and white colonies were selected as positive for the insertion.

Selected colonies were grown in LB supplied with 100 ng/μL of ampicillin. Tubes were placed in a shaker at 200 rpm ON (minimum of 12 h) at 37 °C. Plasmids were extracted from concentrated bacterial cultures using the QIAprep Spin Miniprep Kit (QIAGEN, Venlo, The Netherlands) and concentrations were measured using a Nanodrop ND-1000 spectrophotometer (Thermo Fisher Scientific, Waltham, MA, USA). To confirm the identity of the cloned products, inserts were reamplified from plasmids using the same conditions previously mentioned, and resequenced using 575 ng of plasmid and the generic T7 and Sp6 primers matching the vector.

Five nanograms of DNA from eight samples of each of the 14 cultivars, and a 1:10 8-serial dilution (5 to 5 × 10^−7^ ng) of each plasmid with the different TE family inserts, were used for qRT-PCR with 5 μL of SYBR green (Molecular Probes – Thermo Fisher Scientific, Waltham, MA, USA) and 2.5 μL of the mixed primer pair (3.2 μM), in a 10 μL reaction (three technical replicates per each sample or dilution); three additional reactions with water instead of DNA were used as controls in each primer tested (these showed no amplicons after reactions were completed). Samples were aliquoted in 384-well plates using a Biomek 3000 Laboratory Automation System (Beckman Coulter, Brea, CA, USA), and the qRT-PCR was run using a QuantStudio 6 Flex Real-Time PCR system (Applied Biosystems-Life Technologies, Carlsbad, CA, USA). Cycling conditions were 94 °C for 2 min, followed by 35 cycles of 94 °C for 30 s, 60 °C for 30 s and 72 °C for 45 s. A melting curve stage was added: 95 °C for 15 s, 60 °C for 1 min and 95 °C for 15 s.

To find out the molecule copy number (mcn) in the dilution series, we used the amount of DNA from each point of the serial dilution and the size of the plasmid plus insert [92], and performed a log_10_ transformation. The standard curve was built by plotting the C_t_ values (average of technical replicates) against the log10 of mcn. The linear equation for the slope y = mx + b was used to determine the log_10_ mcn intercepts (x) for the C_t_ values of the eight replicates in each of the 14 cultivars for all primers tested. The log_10_ mcn values where then back-transformed using the power function (10^x^). The same procedure was conducted to find out the copy numbers for the reference gene. The copy numbers of the reverse transcriptases of each family for each sample were normalized to the copy numbers of the reference gene (ETIF1) and the average absolute copy number and standard deviation (from the 8 replicates), were calculated and plotted for each cultivar and TE family.

Statistical differences for each TE copy number among cultivars, were determined using the non-parametric test of Kruskal-Wallis, followed by multiple comparisons using Dunn’s test, using GraphPad Prism version 6.0 (GraphPad Software, La Jolla California USA). Correlation coefficients were calculated in excel for the relationship between the expected copy number of TEs in each family estimated using the primer pairs to BLAST against the flax genome, and either, the calculated copy numbers from qPCR, or the number of scored bands in the SSAPs.

### Sequence-Specific Amplification Polymorphism (SSAP)

One hundred nanograms of each DNA sample were used for restriction digestion at 37 °C for 16 h, with 10 units of *Eco*RI and supplemented with 0.03 mg of BSA and 1× restriction-ligation buffer (10 μL of the digestion were used to check the restriction in a 1% agarose gel). A 1 μM mixture of *Eco*RI adapter 1 and 2 (Additional file 1B), were ligated to the digested ends for 16 h, using 0.2 mM ATP, 1× restriction-ligation buffer and 0.004 units of T4 DNA ligase (Invitrogen, Carlsbad, CA, USA). Ligations were centrifuged and diluted with 80 μL of 1× TE.

To confirm ligation efficiency, a cold PCR (with non-radioactively labelled TE primer) was performed using 1× Taq Buffer, 2 mM MgCl_2_, 0.2 mM of each dNTP, 0.4 μM of the specific TE primer and 0.4 μM of the EcoRI primer 00 (Additional file 1B), 1.5 units of recombinant Taq DNA polymerase (Thermo Fisher Scientific, Waltham, MA, USA) and 5 μL of the diluted restriction-ligation. Cycling conditions were 94 °C for 5 min, followed by 35 cycles of 94 °C for 30 s, 56 °C for 30 s and 72 °C for 1 min, finalizing with an extension at 72 °C for 5 min.

Retrotransposon primer labelling with P^33^ was performed using the LTR TE specific primers (Additional file 1B) at a final concentration of 4 μM, 1× kinase buffer A, 0.5 units of T4 kinase (Thermo Fisher Scientific, Waltham, MA, USA) and 1 μCi of gamma ATP^33^ (PerkinElmer Health Sciences, Boston, MA, USA). The cycling conditions to label the primer were: 1 h at 37 °C and 15 min at 70 °C to inactivate the kinase enzyme (the oligo was kept at −20 °C until used in the SSAP PCR).

SSAP PCR was performed with 1× buffer, 2 mM MgCl_2_, 0.2 mM of each dNTP, 0.4 μM of adapter primer, 0.16 μM of specific radioactively labeled primer, 1.5 units of recombinant Taq DNA polymerase (Thermo Scientific, Thermo Fisher Scientific, Waltham, MA, USA) and 2.5 ng of the restriction-ligation product. Cycling conditions were as following: 94 °C for 5 min followed by 13 cycles of 94 °C for 30 s, 65 °C for 30 s and 72 °C for 2 min; then 25 cycles of 94 °C for 30 s, 56 °C for 30 s and 72 °C for 2 min; finishing at 72 °C for 10 min. After PCR the product was diluted 1:1 with 2× AFLP loading buffer and kept at −20 °C until running the gel. PCR products were separated in 6% denaturing polyacrylamide gels on a Bio-Rad Sequi-Gen GT electrophoresis system (Bio-Rad, Hercules, CA, USA). After the run, the gel was dried and adhered to Whatman paper, and exposed from 1 to 3 days to Kodak Biomax XAR films (Carestream Health Inc., Rochester, New York, USA) and then developed for band scoring.

### Band scoring and maximum likelihood (ML) tree

Exposed films displaying the SSAP band patterns were captured as images (.tif files) and used as input in GelAnalyzer [93] where bands were digitally scored as present = 1 or absent = 0. The scored bands were used to create a binary matrix that was formatted as a nexus file, and utilized as input to generate a maximum likelihood tree using IQ-TREE v1.4.4 [46]. The parameters used were: −m TEST (to test for an optimal substitution model of evolution) and –b 1000 (to do 1000 bootstrap assays). The general time reversible model for binary data was selected to produce a consensus ML tree which was visualized an formatted in MEGA v6.06 [85].

### Band recovery and sequencing

To recover the polymorphic bands, the exposed film was overlaid on the original dried gels on Whatman (both film and gel were pinned previously on the corners to allow matching). A clean scalpel was used to cut the mapped band on surface of the gel-Whatman assembly, and the detached piece was placed in a 1.5 mL tube with 35 μL of nuclease free water. The band in water was vortexed for 1 min and spun down for incubation at 37 °C for 15–16 h. The liquid was recovered to a new 1.5 mL tube and 5 μL were used for a PCR with 1× Taq buffer, 2 mM of MgCl_2_, 0.2 mM dNTPs, 0.2 μM of each primer and 1 unit of recombinant Taq polymerase (Thermo Fisher Scientific, Waltham, MA, USA). Primers for the PCR corresponded to the LTR specific primer for the band along with the *Eco*RI adapter primer (Additional file 1B). Cycling conditions were as following: 94 °C for 2 min followed by 35 cycles of 94 °C for 30 s, 56 °C for 30 s and 72 °C for 2 min, and a final extension at 72 °C for 10 min. The total PCR (25 μL) was run on a 1% agarose gel at 80 V for 60 min and the bands were eluted using the Wizard SV gel and PCR clean-up system (Promega, Madison, WI, USA). Eluted products were quantified using a Nanodrop ND-1000 spectrophotometer (Thermo Fisher Scientific, Waltham, MA, USA). For sequencing, 75 to 225 ng of the eluted product was used (depending on the band size) along with a primer at a final concentration of 0.25 μM (forward or reverse primers corresponded to the same primers used for PCR). Sequencing reactions were performed with the BigDye terminator v3.1 cycle sequencing kit (Applied Biosystems - Thermo Fisher Scientific, Waltham, MA, USA) using a 3730 Genetic Analyzer equipment (Applied Biosystems -Thermo Fisher Scientific, Waltham, MA, USA).

Sections corresponding to the LTRs from sequenced SSAP bands were first compared to the corresponding LTR reference sequence used for primer design (Additional file 5) to see if amplification matched the expected family. Since some LTR variation exists between members of the same family we allowed an identity of over 90% for matches between reference and sequenced bands for all families, with the exception of family RLC_Lu6 were identity of LTRs from the reference Ty1-*copia* element was the lowest (Additional file 2), and an 80% identity was allowed). Then, the complete sequence including LTR plus flanking region was compared to the flax genome deposited in phytozome [94, 95] using blastn and Gbrowse to determine the insertion site of the TE. Accepted blastn results bore 100% identity (or close to 100% - sequencing errors might change a few bases) for the full sequenced band (LTR+flanking region) when the band was initially present (SSAP) in the cultivar Bethune, which represents the reference genome. When the band was not present in this cultivar then the similarity to the LTR and the flanking region was found in different sections of the genome as expected.

Once mapped on the genome, the IDs of the flax genes with associated TE insertions, were used to find the closest *Arabidopsis thaliana* ortholog from a database previously obtained by performing blast analysis of flax transcripts against the peptide TAIR database (release 10). Arabidopsis ortholog IDs were then used to perform functional Gene Ontology (GO) classification using the GO annotation search from TAIR [96], to find out what categories of genes were predominantly affected by TE insertions. An additional enrichment analysis was performed using AgriGO [97, 98] by comparing the Arabidopsis orthologs to the background of all Arabidopsis genes using a Fisher test, a Yekutieli multiple test adjustment and a minimum of 1 mapping read.

### Validation of TE insertions

Once the TE insertions were mapped to the genome, primers were designed from the flanking regions of the transposable element insertion site to validate the polymorphism encountered with the initial SSAP gels. For the TEs that fell within genes we looked for paralogs and performed an alignment to select allele-specific primers which would not bind related genes. Twenty eight primers were designed with Primer3 [89, 90] under the same parameters cited above to be compatible with the original LTR-derived primers, but with a product size range of 200–1000 bp (Additional file 1C), and were used to perform amplification in eight replicate plants in each one of the 14 cultivars. Five nanograms of DNA from each of eight samples per cultivar was used for PCR on 384-well plates using 1× Taq buffer, 2 mM of MgCl_2_, 0.2 mM dNTPs, 0.2 μM of each primer and 1 unit of recombinant Taq polymerase (Thermo Scientific - Thermo Fisher Scientific, Waltham, MA, USA) in a 10 μL reaction. Cycling conditions were as following: 94 °C for 2 min followed by 35 cycles of 94 °C for 30 s, 60 °C for 30 s (this temperature varied according to the primer used – see Additional file 1C) and 72 °C for 1 min, with a final extension at 72 °C for 5 min. Bands were visualized in 1% agarose gels run at 90 V for 60 min.

### Expression of genes with TE insertions

We selected four genes to test their expression in five cultivars that were polymorphic for the respective TE insertion (Fig. 4). The primer pairs per gene were named according to their gene of origin: Pyruvate carboxylase (PYR), Rabgap/TBC domain containing protein-1 (RAB1), Laccase-13-related (LAC), and Rabgap/TBC domain containing protein-2 (RAB2) (Fig. 4 and Additional file 1D).

cDNA from three tissues (leaf + apical meristem, stem and roots) from four biological replicates (different plants), was used to evaluate the primer pairs of each gene using qRT-PCR. Seven reference genes were tested for stability among tissues and replicates [91] (Additional file 1E). While all seven genes were stable, the three with higher stability according to Bestkeeper [99] and GeNorm [100] were GAPDH (glyceraldehyde 3-phosphate dehydrogenase), ETIF5A (eukaryotic translation initiation factor 5 A), and EF1A (elongation factor 1-α). These were used to generate the geometric mean for relative quantification of the test genes using the ΔCt of the reference – the test gene. Statistical differences in each gene among cultivars were calculated using unpaired two-tailed *t*-tests after a Bonferroni correction for multiple comparisons (*p* < 0.005), using GraphPad Prism version 6.0 (GraphPad Software, La Jolla California USA).

Samples were aliquoted in 384-well plates (with three technical replicates per sample and tissue combination) using a Biomek 3000 Laboratory Automation System (Beckman Coulter, Brea, CA, USA), and the qRT-PCR was run using a QuantStudio 6 Flex Real-Time PCR system (Applied Biosystems-Life Technologies, Carlsbad, CA, USA). Sample reactions were done in 10 μL with 5 μL of SYBR-green (Molecular Probes – Thermo Fisher Scientific, Waltham, MA, USA), 2.5 μL of the mixed primer pair (3.2 μM) and 2.5 μL of a 1:50 dilution of the synthesized cDNA. Cycling conditions were: 95 °C for 2 min followed by 40 cycles of 95 °C for 30 s, 60 °C for 1 min. A melting curve stage was added: 95 °C for 15 s, 60 °C for 1 min and 95 °C for 15 s.

## Additional files

Additional file 1: (A-E). Primers. Sets of primers for all experiments performed. (XLSX 18 kb)
Additional file 2: Insertion age and domains of representative sequences from selected Ty1-*copia* families. Characterization of six Ty1-*copia* elements which represent the six families investigated in this study. (XLSX 11 kb)
Additional file 3: SSAP example of retrotransposon family RLC_Lu1. The SSAP was run for cultivars: 1. Bethune, 2. Lutea, 3. Stormont Cirrus, 4. Adelie, 5. Aurore, 6. Belinka, 7. Blizzard, 8. Drakkar, 9. Evea, 10. Hermes, 11. Oleane, 12. Oliver, 13. *rdf*, 14. Violin. (PPTX 606 kb)
Additional file 4: Mapping of insertion sites of SSAP bands sequenced. (DOC 187 kb)
Additional file 5: Sequences from SSAP eluted bands. The LTR sequences from representative members of the six TE families investigated are presented along the sequenced sections of the SSAP bands (Genbank accession numbers: KX364308 to KX364373). Boxed sequences correspond to the LTR of representative elements of each TE family (Additional file 2), in direct and in reverse orientation; some LTR boundaries were adjusted from the original predictions of LTR finder (Additional file 2) after mapping analysis. The LTR primer region is shown in blue. The sequenced LTR region present in SSAP bands is shown in green. The polymorphic bands are named according to the original number given when eluting the band from the gel and match Additional file 4 descriptions. Sequences of the EcoRI adaptor primer and of the LTR primer have been trimmed from the sequences. Names of the TEs are explained in the Methods section. (DOCX 33 kb)
Additional file 6: Comparison of selected SSAP band scores and PCR validation in 14 flax accessions. SSAP band polymorphisms were selected for validation using conventional PCR (see Methods). 1 = present, 0 = absent, W = weak band at expected size, (?) = weak band at non-expected size, 1+L = expected band plus an additional lower band, 1+H = expected band plus an additional higher band, P = polymorphic among replicates of same cultivar. Colors from accessions represent flax types as in Fig. 3. (DOCX 41 kb)
Additional file 7: GO functional categories of flax closest orthologs in Arabidopsis. The flax gene sequences from Additional file 4 where used to search the Arabidopsis closest ortholog, and these were used for functional classification using Gene Ontology (see Methods). (XLSX 9 kb)

## Abbreviations

ACS: Aminocyclopropane-carboxylate synthase
CDC: Crop Development Center
EF1A: Elongation factor 1-α
ETIF1: Eukaryotic translation initiation factor 1
ETIF3E: Eukaryotic translation initiation factor 3 E
ETIF5A: Eukaryotic translation initiation factor 5 A
GAG: Group-specific antigen
GAPDH: Glyceraldehyde 3-phosphate dehydrogenase
GO: Gene Ontology
INRA: National Institute for Agronomic Research (Institut National de la Recherche Agronomique)
INT: Integrase
IRAP: Inter-Retrotransposon Amplified Polymorphism
LAC: Laccase
LTR: Long Terminal Repeat
Lu: *Linum usitatissimum*
mcn: Molecule copy number
MITE: Miniature Inverted-repeat TE
ML: Maximum likelihood
MULE: Mu-like Element
MYB: Myeloblastosis
PLR: Pinoresinol-lariciresinol reductase
PR: Protease
PYR: Pyruvate carboxylase
qPCR: Quantitative PCR
RAB: Rabgap/TBC domain containing protein
*rdf*: Reduced fiber
RLC: Retrotransposon-LTR-Copia
RNAse H/RH: Ribonuclease H
RT: Reverse transcriptase
SSAP: Sequence-Specific Amplification Polymorphism
TD: Transposon display
TE: Transposable element
VRN: Vernalization
