# Supplementary material for: Ty1-copia elements reveal diverse insertion sites linked to polymorphisms among flax (Linum usitatissimum L.) accessions
Source: BMC Genomics. 2016 Dec 7;17:1002. doi: 10.1186/s12864-016-3337-3 (PMC5142383; doi:10.1186/s12864-016-3337-3)
Supplement: Additional file 3: — SSAP example of retrotransposon family RLC_Lu1. The SSAP was run for cultivars: 1. Bethune, 2. Lutea, 3. Stormont Cirrus, 4. Adelie, 5. Aurore, 6. Belinka, 7. Blizzard, 8. Drakkar, 9. Evea, 10. Hermes, 11. Oleane, 12. Oliver, 13. rdf, 14. Violin. (PPTX 606 kb) [file 12864_2016_3337_MOESM3_ESM.pptx]

## Slide 1
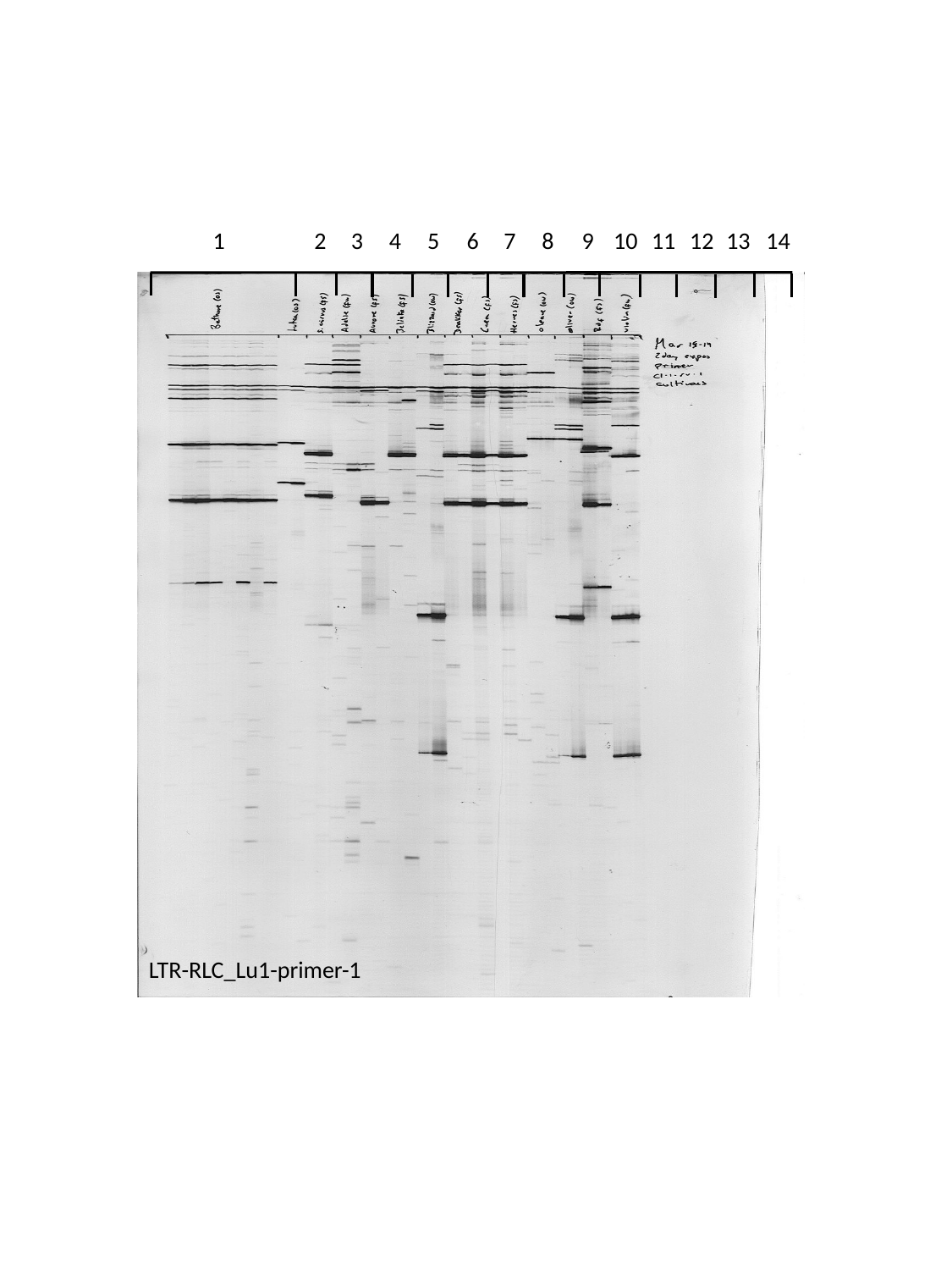

3
5
7
9
10
12
1
14
2
4
6
8
11
13
LTR-RLC_Lu1-primer-1

## Slide 2
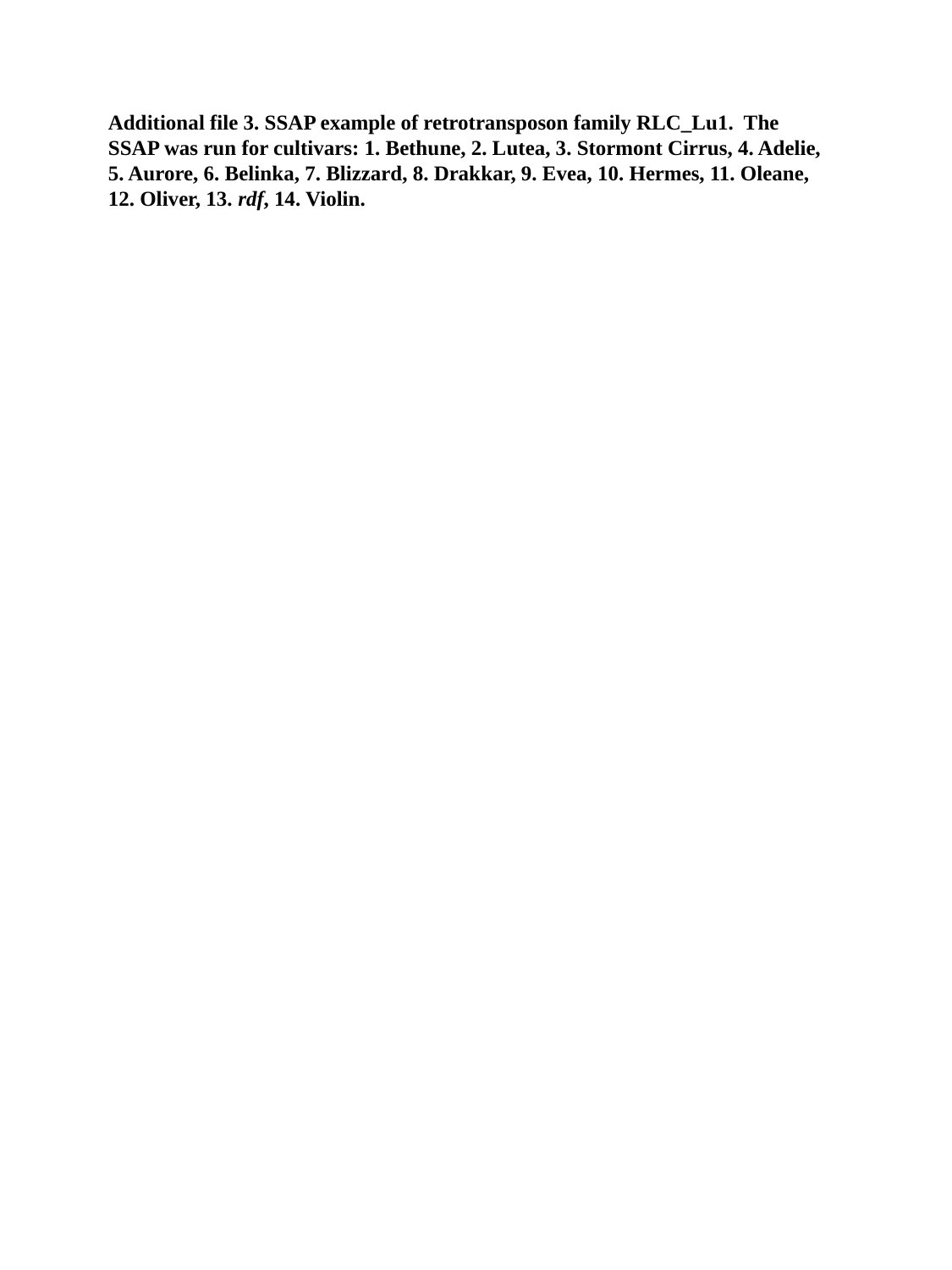

Additional file 3. SSAP example of retrotransposon family RLC_Lu1. The SSAP was run for cultivars: 1. Bethune, 2. Lutea, 3. Stormont Cirrus, 4. Adelie, 5. Aurore, 6. Belinka, 7. Blizzard, 8. Drakkar, 9. Evea, 10. Hermes, 11. Oleane, 12. Oliver, 13. rdf, 14. Violin.
