# Supplementary material for: Ty1-copia elements reveal diverse insertion sites linked to polymorphisms among flax (Linum usitatissimum L.) accessions
Source: BMC Genomics. 2016 Dec 7;17:1002. doi: 10.1186/s12864-016-3337-3 (PMC5142383; doi:10.1186/s12864-016-3337-3)
Supplement: Additional file 4: — Mapping of insertion sites of SSAP bands sequenced. (DOC 187 kb) [file 12864_2016_3337_MOESM4_ESM.doc]

**Additional file 4.** Mapping of insertion sites of SSAP bands sequenced

| **IDs** | | **OS** | | | **OW** | | | **FW** | | **FS** | | | | | | **Annotation** | | |
| --- | --- | --- | --- | --- | --- | --- | --- | --- | --- | --- | --- | --- | --- | --- | --- | --- | --- | --- |
| **Copia family primer** | **band ID^1^** | **rdf** | **bet** | **lut** | **ole** | **bli** | **oli** | **vio** | **ade** | **sci** | **eve** | **dra** | **her** | **bel** | **aur** | **Phytozome match** | **Insertion site** | **Orientation gene / TE^4^** |
| LTR-RLC_Lu0-primer3 | 5 |  |  |  |  |  |  |  |  |  |  |  |  |  |  | Lus10014555 (NADH:ubiquinone reductase (non-electrogenic) / Ubiquinone reductase) | Intron 2 | Same |
|  | 6 |  |  |  |  |  |  |  |  |  |  |  |  |  |  | Lus10013587 (Protein virilizer homolog) | Intron 4 | Same |
|  | 7 |  |  |  |  |  |  |  |  |  |  |  |  |  |  | Lus10022077 (Pyruvate carboxylase) | Exon 8 | Same |
|  | 8 |  |  |  |  |  |  |  |  |  |  |  |  |  |  | Lus10036500 (Rabgap/TBC domain containing protein) | Intron 6 | Same |
|  | 10 |  |  |  |  |  |  |  |  |  |  |  |  |  |  | Intergenic | Intergenic | N/A |
|  | 11 |  |  |  |  |  |  |  |  |  |  |  |  |  |  | Lus10043191 (Pyruvate dehydrogenase E1 component subunit beta, mitochondrial) | Intron 19 | Opposite |
|  | 12 |  |  |  |  |  |  |  |  |  |  |  |  |  |  | Lus10041231 (RRP12-like protein) | Intron 1 | Opposite |
|  | 14 |  |  |  |  |  |  |  |  |  |  |  |  |  |  | Lus10017623 (DNA ligase) | Intron 5 | Opposite |
|  | 16 |  |  |  |  |  |  |  |  |  |  |  |  |  |  | Lus10033880 (Transcription factor EMB1444-related) | Intron 5 | Same |
|  | 17 |  |  |  |  |  |  |  |  |  |  |  |  |  |  | Lus10009307 (Protein T01H10.8) | Intron 4 | Opposite |
| LTR-RLC_Lu1-primer1 | 12 |  |  |  |  |  |  |  |  |  |  |  |  |  |  | Intergenic | Intergenic | N/A |
|  | 13 |  |  |  |  |  |  |  |  |  |  |  |  |  |  | Lus10033319 (Zinc finger, ZZ type (ZZ) // TAZ zinc finger (zf-TAZ) // Histone acetylation protein (HAT_KAT11)) | Intron 12 | Opposite |
|  | 14 |  |  |  |  |  |  |  |  |  |  |  |  |  |  | Intergenic | Intergenic | N/A |
|  | 15 |  |  |  |  |  |  |  |  |  |  |  |  |  |  | Lus10001114 (Alpha/beta hydrolases superfamily family) | Intron 3 | Same |
|  | 16 |  |  |  |  |  |  |  |  |  |  |  |  |  |  | Lus10016813 (Mitofilin). | Intron 4 | Same |
|  | 18 |  |  |  |  |  |  |  |  |  |  |  |  |  |  | Lus10026400 (Laccase-13-related) | Exon 3 | Opposite |
|  | 19 |  |  |  |  |  |  |  |  |  |  |  |  |  |  | Lus10040349 (Rabgap/TBC domain containing protein) | Intron 4 | Opposite |
|  | 21 |  |  |  |  |  |  |  |  |  |  |  |  |  |  | Lus10019489 (Telomere-length maintenance and DNA damage repair (TAN)) | Intron 38 | Opposite |
|  | 22 |  |  |  |  |  |  |  |  |  |  |  |  |  |  | Lus10030711 (Spatacsin) | Intron 3 | Same |
|  | 23 |  |  |  |  |  |  |  |  |  |  |  |  |  |  | Lus10022840 (RNI-like superfamily protein) | Intron 7 | Same |
|  | 24 |  |  |  |  |  |  |  |  |  |  |  |  |  |  | The LTRs and an apparent degenerate internal TE region overlap a section from exon 3 to exon 5 of gene Lus10036612 (Formin-like protein 13)^3^ | Exon 3 | Opposite |
|  | 25 |  |  |  |  |  |  |  |  |  |  |  |  |  |  | Lus10001216 (Cytoskeleton-associated protein 5), is 64 bp from TE^2^ | Downstream of gene | Opposite |
|  | 26 |  |  |  |  |  |  |  |  |  |  |  |  |  |  | Lus10034176 (anaphase-promoting complex subunit 5) | Intron 7 | Same |
| LTR-RLC_Lu1-primer2 | 9 |  |  |  |  |  |  |  |  |  |  |  |  |  |  | Lus10035905 (DEK protein) | Intron 3 | Opposite |
|  | 12 |  |  |  |  |  |  |  |  |  |  |  |  |  |  | Lus10025751 (Ubiquitin carboxyl-terminal hydrolase) | Intron 5 | Same |
|  | 14 |  |  |  |  |  |  |  |  |  |  |  |  |  |  | Lus10036170 (Tousled-like protein kinase) | Intron 15 | Opposite |
|  | 15 |  |  |  |  |  |  |  |  |  |  |  |  |  |  | Lus10026982 (Exocyst complex component 3) | Intron 19 | Same |
| LTR-RLC_Lu2-primer1 | 2 |  |  |  |  |  |  |  |  |  |  |  |  |  |  | Lus10030545 (CBL-interacting serine/threonine-protein kinase 2) is 5 bp from TE^2^ | Downstream of gene | Opposite |
|  | 4 |  |  |  |  |  |  |  |  |  |  |  |  |  |  | Lus10027426 (N-methylcoclaurine 3'-monooxygenase / N-methylcoclaurine 3'-hydroxylase) | Exon 2 | Same |
|  | 5 |  |  |  |  |  |  |  |  |  |  |  |  |  |  | Lus10040443 (Pinoresinol-lariciresinol reductase 3-related) | Exon 2 | Same |
|  | 6 |  |  |  |  |  |  |  |  |  |  |  |  |  |  | Intergenic | Intergenic | N/A |
|  | 7 |  |  |  |  |  |  |  |  |  |  |  |  |  |  | Lus10020601 (DNA-directed RNA polymerase II protein) is 295 bp from TE^2^ | Upstream of gene | Same |
|  | 9 |  |  |  |  |  |  |  |  |  |  |  |  |  |  | Lus10029082 (Uncharacterized protein) is 990 bp from TE^2^ | Downstream of gene | Opposite |
|  | 10 |  |  |  |  |  |  |  |  |  |  |  |  |  |  | TE is between genes Lus10035815 (PPR repeat (PPR) // PPR repeat family (PPR_2) // DYW family of nucleic acid deaminases (DYW_deaminase)) and Lus10035816 (RNA polymerase II transcription elongation factor Elongin/SIII, subunit elongin B) 178bp and 749 bp from them^2^ | Upstream of two genes | TE opposite to first gene and in same orientation of second gene |
|  | 12 |  |  |  |  |  |  |  |  |  |  |  |  |  |  | Intergenic | Intergenic | N/A |
|  | 13 |  |  |  |  |  |  |  |  |  |  |  |  |  |  | Lus10001212 (Transcription factor Tfb2 (Tfb2) // Protein tyrosine kinase (Pkinase_Tyr)) | Intron 2 | Same |
| LTR-RLC_Lu6-primer3 | 5 |  |  |  |  |  |  |  |  |  |  |  |  |  |  | Intergenic | Intergenic | N/A |
|  | 8 |  |  |  |  |  |  |  |  |  |  |  |  |  |  | The complete 3’ LTR and a partial degenerate 5’ LTR from this element flank Lus10037467 (F-box domain protein), and a RNAse H domain was identified close to the 3’ LTR; the element is also placed 13 bp from Lus10037468 (Domain of unknown function (DUF966))^2^ | Containing one gene and downstream from another | TE opposite to gene inside and in same orientation of second gene |
|  | 9 |  |  |  |  |  |  |  |  |  |  |  |  |  |  | Intergenic | Intergenic | N/A |
|  | 13 |  |  |  |  |  |  |  |  |  |  |  |  |  |  | The complete LTR overlaps intron 1 and exon 1 of gene Lus10013474 (Uncharacterized protein). The 5kb upstream of the 3’LTR give no indication of a complete TE so this could be a solo LTR^2,3^ | Intron 1 | Opposite |
|  | 15 |  |  |  |  |  |  |  |  |  |  |  |  |  |  | Intergenic | Intergenic | N/A |
|  | 16 |  |  |  |  |  |  |  |  |  |  |  |  |  |  | Intergenic | Intergenic | N/A |
| LTR-RLC_Lu8-primer1 | 4 |  |  |  |  |  |  |  |  |  |  |  |  |  |  | Intergenic | Intergenic | N/A |
|  | 5 |  |  |  |  |  |  |  |  |  |  |  |  |  |  | Lus10036983 (ATP dependent RNA Helicase) | Exon 1 | Same |
|  | 6 |  |  |  |  |  |  |  |  |  |  |  |  |  |  | Lus10020564 (Uncharacterized protein) | Exon 1 | Same |
|  | 7 |  |  |  |  |  |  |  |  |  |  |  |  |  |  | Lus10028760 (1-aminocyclopropane-1-carboxylate synthase 2-related) | Intron 2 | Same |
|  | 9 |  |  |  |  |  |  |  |  |  |  |  |  |  |  | Lus10030545 (CBL-interacting serine/threonine-protein kinase 2) is 721 bp from TE^2^ | Upstream of gene | Same |
|  | 10 |  |  |  |  |  |  |  |  |  |  |  |  |  |  | Lus10016251 (TATA box-binding protein associated factor RNA polymerase I subunit B) | Unique exon | Opposite |
|  | 11 |  |  |  |  |  |  |  |  |  |  |  |  |  |  | The 5’LTR overlaps with exon 3 of Lus10039295 (WRKY transcription factor 27-related)^3^ | Exon 3 | Same |
|  | 12 |  |  |  |  |  |  |  |  |  |  |  |  |  |  | Lus10029998 (Clathrin coat assembly protein AP180) | Exon 1 | Same |
|  | 13 |  |  |  |  |  |  |  |  |  |  |  |  |  |  | Lus10009285 (GOS-28 Snare-related) is 7 bp from a partial section of TE LTR^2^ | Upstream of gene | Opposite |
|  | 14 |  |  |  |  |  |  |  |  |  |  |  |  |  |  | Lus10014756 (Polynucleotide 5’-hydroxyl-kinase NOL9) | Intron 9 | Opposite |
|  | 15 |  |  |  |  |  |  |  |  |  |  |  |  |  |  | Lus10031899 (Tetratricopeptide-like helical) | Exon 1 | Same |
|  | 16 |  |  |  |  |  |  |  |  |  |  |  |  |  |  | Lus10001449 (Glycerol-3-phosphate acyltransferase 2-related) is 349 bp from TE^2^ | Downstream of gene | Same |
|  | 17 |  |  |  |  |  |  |  |  |  |  |  |  |  |  | Lus10038912 (IRE Serine/threonine protein kinase) | Exon 3 | Opposite |
|  | 18 |  |  |  |  |  |  |  |  |  |  |  |  |  |  | Lus10033384 (Protein kinase domain (Pkinase) // Leucine rich repeat N-terminal domain (LRRNT_2) // Leucine rich repeat (LRR_8)) | Exon 1 | Same |
| LTR-RLC_Lu28-primer1 | 1 |  |  |  |  |  |  |  |  |  |  |  |  |  |  | Lus10037058 (Neurolysin / neurotensin endopeptidase) | Exon 8 | Opposite |
|  | 4 |  |  |  |  |  |  |  |  |  |  |  |  |  |  | Lus10017405 (AAA-type ATPase domain-containing protein related) | Intron 16 | Same |
|  | 6 |  |  |  |  |  |  |  |  |  |  |  |  |  |  | Lus10041785 (Uncharacterized gene) is 574 bp from TE^2^ | Downstream of gene | Opposite |
|  | 7 |  |  |  |  |  |  |  |  |  |  |  |  |  |  | Lus10008548 (Variant SH3 domain protein (SH3_9)). | Intron 14 | Opposite |
|  | 9 |  |  |  |  |  |  |  |  |  |  |  |  |  |  | Intergenic | Intergenic | N/A |
|  | 11 |  |  |  |  |  |  |  |  |  |  |  |  |  |  | Lus10001366 (Disease resistance protein related). | Intron 1 | Same |
|  | 12 |  |  |  |  |  |  |  |  |  |  |  |  |  |  | Lus10025655 (ARM repeat superfamily protein) | Intron 18 | Opposite |
|  | 14 |  |  |  |  |  |  |  |  |  |  |  |  |  |  | Lus10037943 (Limit dextrinase / R-enzyme) | Intron 5 | Same |
|  | 20 |  |  |  |  |  |  |  |  |  |  |  |  |  |  | Lus10001455 (Transcriptional regulator BRCA1) | Intron 7 | Opposite |
|  | 22 |  |  |  |  |  |  |  |  |  |  |  |  |  |  | The 3’LTR overlaps with exon 4 of Lus10008138 (Plant protein of unknown function DUF936). The 5’LTR is 53 bp from Lus10081137 (Uncharacterized protein)^2.3^ | Upstream of gene | Opposite to both |

Mapping of SSAP polymorphic bands in six *Ty1*-copia families across 14 flax cultivars: rdf (*rdf)*, bet (Bethune), lut (Lutea), ole (Oleane), bli (Blizzard), oli (Oliver), vio (Violin), ade (Adelie), sci (Stormont Cirrus), eve (Evea), dra (Drakkar), her (Hermes), bel (Belinka), aur (Aurore). The presence of the insertion in each cultivar is shown by the shading beneath each accession. The cultivars are classified in four flax types: oil-spring (OS), oil-winter (OW), fiber-winter (FW) and fiber-spring (FS).

^1^Refers to the number of the band identified. Sequences of the respective bands can be found in Additional file 5.

^2^When the TE was not inside an annotated gene the distance to the closest gene(s) was calculated. The distance was recorded if it was within 1kb of the gene. Distances of insertions not present in Bethune (the reference genome) are inferred from the match of the flanking region.

^3^When the TE mapping overlapped with the phytozome annotation the insertion site was annotated as the 5’-most region of the TE.

^4^In this column N/A means non applicable.
