# Supplementary material for: Ty1-copia elements reveal diverse insertion sites linked to polymorphisms among flax (Linum usitatissimum L.) accessions
Source: BMC Genomics. 2016 Dec 7;17:1002. doi: 10.1186/s12864-016-3337-3 (PMC5142383; doi:10.1186/s12864-016-3337-3)
Supplement: Additional file 5: — Sequences from SSAP eluted bands. The LTR sequences from representative members of the six TE families investigated are presented along the sequenced sections of the SSAP bands (Genbank accession numbers: KX364308 to KX364373). Boxed sequences correspond to the LTR of representative elements of each TE family (Additional file 2), in direct and in reverse orientation; some LTR boundaries were adjusted from the original predictions of LTR finder (Additional file 2) after mapping analysis. The LTR primer region is shown in blue. The sequenced LTR region present in SSAP bands is shown in green. The polymorphic bands are named according to the original number given when eluting the band from the gel and match Additional file 4 descriptions. Sequences of the EcoRI adaptor primer and of the LTR primer have been trimmed from the sequences. Names of the TEs are explained in the Methods section. (DOCX 33 kb) [file 12864_2016_3337_MOESM5_ESM.docx]

**Additional file 5. Sequences from SSAP eluted bands. The LTR sequences from representative members of the six TE families investigated are presented along the sequenced sections of the SSAP bands (Genbank accession numbers: KX364308 to KX364373). Boxed sequences correspond to the LTR of representative elements of each TE family (Additional file 2), in direct and in reverse orientation; some LTR boundaries were adjusted from the original predictions of LTR finder (Additional file 2) after mapping analysis. The LTR primer region is shown in blue. The sequenced LTR region present in SSAP bands is shown in green. The polymorphic bands are named according to the original number given when eluting the band from the gel and match Table 3 descriptions. Sequences of the EcoRI adaptor primer and of the LTR primer have been trimmed from the sequences. Names of the TEs are explained in the methods section.**

**RLC_Lu0-primer3 * EcoRI**

>RLC_Lu0-1 (LTR_sense)

TGTTGAATTACAGAAATAACTAGGATATTATTAGGCGTATAACTTGGAGTAATCTAGCTATCCTATAGAATCAGTCTAGCCTGATTTAGGCAGTGTATTCATAGTAAGTGGTAGGTAGGATAAATCCCTGATTTGTAGGGATTACTACGAGCTGGATCTGCATCCTAATGATGCAGACTGTGTATATATGTAAAGGAAACCACAGAAATAAGAATATCATACCAGAATCACTCAGATTTCTGGATTTCTGCA

>RLC_Lu0-1 (LTR_reverse)

TGCAGAAATCCAGAAATCTGAGTGATTCTGGTATGATATTCTTATTTCTGTGGTTTCCTTTACATATATACACAGTCTGCATCATTAGGATGCAGATCCAGCTCGTAGTAATCCCTACAAATCAGGGATTTATCCTACCTACCACTTACTATGAATACACTGCCTAAATCAGGCTAGACTGATTCTATAGGATAGCTAGATTACTCCAAGTTATACGCCTAATAATATCCTAGTTATTTCTGTAATTCAACA

>band_5

GATTAATCCTACCTACCACTTACTATGATTACACTGCCTAAATCAGGCTAGTCTGATTCTATAGGATAGCTAGATTACACTAAGTGATACGCCTAATAATATCCTAGTTATTTCTGTAATTCAACAAAAGCCATCAAAAACAGAAAACTGCAAGTGATAATAGCTGCTGCATCTTGGGAGATTTGAAAACCTCACCTCATTGTAAGGCCAAGTGTAGACCCGTGGAGGCTTCTCTTCTACAGGTGGATTGGGAGCAGCTGCTGCATCAGTCCCGACTGCCTCCGAAGTTACAAATCGAACATGGGAACTCGTGAAGCCAGAATGGAAGGTCCTAAATGGAGTTTGAGCCGAGGAAAATGGGAACAATTTTCCCCTTGACATGCCTGATCAACGAAAAATGATTGCAATACTCAAGACATGCATCAGAAAGTAAAATAAGGCCATGGAAGCAACCAAAACGGAAAAGAACCGAGAAT

>band_6

GATTAATCCTACCTACCACTTACTATGATTACACTGCCTAAATCAGGCTAGTCTGATTCTATAGGATAGCTAGATTACACTAAGTGATACGCCTAATAATATCCTAGTTATTTCTGTAATTCAACACTACGCTGTTTCAACACAATTTCAAAGAGCCACGGTAGGCAACAAACAGTAGTGAATGAGAAGAAAAAGAGACAATTGATAATAGGTAAGAGGATTCGCAGTGCCATAATCACCTGTAAACGCTTGTGTGGTTGGGAAGAGTTCTTGTCGAAACAAACATGTGGGCCGAACATTTCCACCAATTCCTTTGAAGTAGCCAAGTCATCTTCCGATTCTAGAAAACTACCATCCACTAAAACATGGGCCATATCATTTCCTGAAGACTGAAGGAGTCCAAGAAGCTCCTTCTTAGCCTCAGTAATAATATGGGAAAACTCTTCAAACTCATTANATCCGCTTGNNTTA

>band_7

GATTAATCCTACCTACCACTTACTATGATTACACTGCCTAAATCAGGCTAGTCTGATTCTATAGGATAGCTAGATTACACTAAGTGATACGCCTAATAATATCCTAGTTATTTCTGTAATTCAACACAGCCGAACCAACTTCTCCCAACTGTGTGATTCGCAAAATGTTGATTGAGCAACTTATTTGAAAATGTGTTAATTGAAGTACTAGAAGGAAATAAAATAGTCTATTACTATACCAGAGAAATACCTCGGTGAAATAGGACCCATCTTGCTGGTAAGTGGTTGAAACTGTGAAGAGATTTGAGCTGCTAGTACCATATTCATTATCCCATTCAAATACCATGGCCCTTGTGGCATTATGATGAACTCTAAAAGGGGGATGTTTATACCATAACGAGAGTGAACCTGTACTCCGTGGATATATTTCA

>band_8

GATTAATCCTACCTACCACTTACTATGATTACACTGCCTAAATCAGGCTAGTCTGATTCTATAGGATAGCTAGATTACACTAAGTGATACGCCTAATAATATCCTAGTTATTTCTGTAATTCAACACTATTTAATTAAAATAGTACCATTAATCTTGAACTGTTAACCTCAGAGGTTCAACCTCTGAAGACAACATAGAAAAGTATATAAGCTACCTGTTCTTGAAGATCTGGAGCACTATCCATCTCTCCATTCTCTGTCAGACTAATAAGAACATCATCTGAATTAGTAGATCCAGACTCTGAACAAGATAAGTCACCAGATGTTTGCATTCTTCCTTCTACCAACATTGATTTAGGATCATGCTTGAAATTATACAGCTTAGAGGCCAACCCTTTAGAATCTCTCCAAGCTT

>band_10

GATTTATTCTACCTACCACTTACTATGAATACACTGCCTAAATCAGGCTAGACTGATTCTATAGGATAGCTAGATTACTCTAAGTTATACGCCTAATAATATCCTAGTTATTTCTGTAATTCAACAAGTCTCATATAGCAAACATGATCAACCATGATCTCAACAACAAGCAACAGAAAACACTCCACGACTAACAAACTAACGAGGGACCAAAATGTAAACAGATGGAAGTATTGAACTAAAACGACAATCACAACAGACTTGCACAATAAACCAAAACGCACAGTTTCATTAACTTATTAATTCCAACAAAAGTTAAGTAAATAAAATAGTAATACTTACTCTTTGCATCTGGCGTACATACTCGC

>band_11

GATTAATCCTACCTACCACTTACTATGATTACACTGCCTAAATCAGGCTAGTCTGATTCTATAGGATAGCTAGATTACACTAAGTGATACGCCTAATAATATCCTAGTTATTTCTGTAATTCAACACCTCTGCAGAGATATTATATTGTTGTTTATTTAGTATGCATCTATTGTGGCATTCTGTTGTGGAGATTTATGCTTTAGGAAACCTCCTTTTTTATAGTGAATATCTCTACTCACTCCATTCGTTGAATTTAGGCTGGATTTACTGGTATTGGAGTTGGCGCAGCTTATCATGGGCTTAAGCCTATTATC

>band_12

GATTAATCCTACCTACCACTTACTATGATTACACTGCCTAAATCAGGCTAGTCTGATTCTATAGGATAGCTAGATTACACTAAGTGATACGCCTAATAATATCCTAGTTATTTCTGTAATTCAACAGTCTTCAATACCTGATTTTGCTTTTCCACTGCAGTCTACAATATCTGATTTTGCTTCACTAGCTGCTGAAGATGAAAATACAAGGGAAGTAGTTTCACGCAGATTCTTGCAGACAATGAAAATGATTTTGAAGGCTACGAAGCGTGCAGGACAAGCTGGAAGGTCCAA

>band_14

GATTAATCCTACCTACCACTTACTATGATTACACTGCCTAAATCAGGCTAGTCTGATTCTATAGGATAGCTAGATTCACTAAGTGATACGCCTAATAATATCCTAGTTATTTCTGTAATTCAACAATTTAAAAGTAACAGTTTTATGTCTGCCTATCTTACATTTTAGGAGAACAAAAATAGATGCAGTTTGGACGACAAGCTGTGGCTTCTTCTGCTGTTGTTGGATTTCATTTTTACTCAAATTGAAAAGATACTTCAACCTAGTAGA

>band_16

GATTAATCCTACCTACCACTTACTATGATTACACTGCCTAAATCAGGCTAGTCTGATTCTATAGGATAGCTAGATTACACTAAGTGATACGCCTAATAATATCCTAGTTATTTCTGTAATTCAACAGTTCTTACTTTATTTAGTGATCCAAGTTGTACAACTCCATAAGGAACAACAGCTACAACAGCAATTGTCTGCAGATCAATAAAAACAAGCAAAAAACATGAGCATGATATCTCTGCTAAAACAATT

>band_17

GATTAATCCTACCTACCACTTACTATGATTACACTGCCTAAATCAGGCTAGTCTGATTCTATAGGATAGCTAGATTACACTAAGTGATACGCCTAATAATATCCTAGTTATTTCTGTAATTCAACATTTTTTTGTTGGAAATAGGAAACACAAGATAGAAGTAAAATAAACATGACATAACTGTCCTTTTGTGGAGCGTAACT

**RLC_Lu1-primer1 * EcoRI**

>RLC_Lu1-1 (LTR_sense)

TGAGGAAATCCCGTTCCTTATTTGTATACAGTCAGATCTTATTAGTTTATTTGCTAGTTAGATAGTTACCATATTAGTTCTAGTCCTATCCTAGTTTAGCTAGCAGATCCTATTAAATAGTTAGAGTAGATATTTTCTTACCTAGTCAGTAGGAGGAATGCTGTATATAATAAACCTACGAGACATGAATAAAAGTAATTCATTCTCTCAATCTTCA

> RLC_Lu1-1 (LTR_reverse)

TGAAGATTGAGAGAATGAATTACTTTTATTCATGTCTCGTAGGTTTATTATATACAGCATTCCTCCTACTGACTAGGTAAGAAAATATCTACTCTAACTATTTAATAGGATCTGCTAGCTAAACTAGGATAGGACTAGAACTAATATGGTAACTATCTAACTAGCAAATAAACTAATAAGATCTGACTGTATACAAATAAGGAACGGGATTTCCTCA

>band_12

TAGGTAAGAAAATATCTACTCTAACTATTTAATAGGATCTGCTAGCTAAACTAGGATAGGACTAGAACTAACATGGTAACTATCTAACTAGCAAATAAACTAATAAGATCTGACTGTATACAAATAAGGAACGGGATTTCCTCAGAACTACATATGTATTACTGGATCTTAGTGGACTCCTTTTTCTATATTTATATATATACCCTTCACGTCTTCCTTGTAGCTTTCATGGATTGGCTGAATTACATGTTAGCCAGGCATTATCCACTCCCATTGTAATTTGTAAGTGGGTTCATCTTTGGAGCATTGTTATAGAGACTATAGCATGTGGTTGTGGTACATTCATGAGAATTGTAGTAACTGCTTTTTTTTTTCACTACCTGTTTATTGGTGGCTTTTCTGCCAGTGGCTTAATATCTAAGCAATGTGAAGTCTGCTTGCTTCATACGGACTTGCTTTCGTTACTCTGCTGAAGATGGATTACTGTTATATTTGCGAGCATACAAGACAATCATGCTCTGGTTTGATGTATTATGTCTGTCTGTATGCTCTTATCTCATAGTTGCGCACAAGGTTTGAAGAATTAGAGAAGTTAAGTGAATGTTGACTGCTACAGTGCTACCTCATATGTTGGTGTCAGTAGTAACTCCGTCCCAGTCCTATCTGTAAGTATGTTGAGCTGA

>band_13

TAGGTAAGAAAATATCTACTCTAACTAATTAATAGGATCTGCTAGTTAAACTAGGATAGGACTAGAACTAATATGGTAACTATCTAACTAGCAAATAAACTAATAAGATCTGACTGTATACACATAAGGAACGGGATTTCCTCACTATATAACCTTTAAGGCTAATTTTTTGTTCAACATTTCTCTTCTTGAATTTGTGATGTTATTGTGGGTTTTTTTGTTGGGCATTCATTTGCTTCTCAATCATCTTGGATGGTTAAAAACTGTGATCGTTAATAATTCTCTAACCTATGATTTTGACGTATTTGCTTTCATACTGTTGAAGGTGTTATGAAGCTGAACAGAAGCGTGAGGAGCGAGAAAGGCATCCTGTGAACCACCGGGAGAAACATGGACTCTATCCGGTAAGTTTGCAACAATTTGTTGTTGGTTGAAACTTTTCTCTCGTTACGATGAACTGTAAACAGCATGACTGGAAATTTTTGAAGGTTGAAATCGGTGATGTGACTGTTGATACGAAGGACCAAGACGAAATTCTTGAGAGT

>band_14

ACTCTACTATTTATAGGATCTGCTAGCTAAACTAGGATAGGACTAGAACTAATATGGTAACTATCTAACTAGCAAATAAACTAATAAGATCTGACTGTATACACATAAGGAACGGGATTTCCTCAATAACCTTTATGATGATCGATATATCCAATAAATACACATTGAGTTTTAATGGTCAACTTAGTTTGATCTTTCTTGGGGAAGAACACAAAAAAGGTGCAACTAAAGACCCTTAGTCAAGTGTCATCCGAAGGACATCCATTAAAACTTTAAAAGGTGACTGACTCTGTAGAACCAGCGTAGGTTGAAGATTCACAAGATAAACCATGGTATGAACCTTTTCGACCCTTAATTGTGAAGGACCCTGAGATTCGAGTAAGAGAGCTCGTGTACGATCCAAAACATGACGATGCTTACACTCTACAAACCATTTTGTTCAAACACACCCGGACAACAATCTTGGAACAGGATACCATTTTCTCAAAAATATAGATGAAGAGCATGCGAGGT

>band_15

TAGGTAAGAAAATATCTACTCTAACTATTTAATAGGATCTGCTAGCTAAACTAGGATAGGACTAGAACTAATATGGTAACTATCTAACTAGCAAATAAACTAATAAGATCTGACTGTATACAAATAAGGAACGGGATTTCCTCAGGATCAAAAACCTCTCAACAGTGATACATCATGACAAAGAACGAGTTACTCTTACGCAGATTATGCAACATGTTTTCCATCAGATACACAAAATTCAATAACAAGGATGTTCAAGTTACCTGTTTCCATGGCAATAAATTACACAGGGCAGAGGCTTTTCTCCAGGACTGACGACGGGCAAATAATGGCTGCATTGAAGAACATCCCCCCTGTCATTTGTTATCTGCATGATATTTGAAATGAAAATATTTAGGAAGGCTAACTAAACTCATCATGCGGGATAAACATGAATATTGTGAATGGTTACCTCCACATCCTTTCTCTGGTACAATTTCCCTCGTAACAT

>band_16

TAGGTAAGAAAATATCTACTCTAACTATTTAATAGGATCTGCTAGCTAAACTAGGATAGGACTAGAACTAATATGGTAACTATCTAACTAGCAAATAAACTAATAAGATCTGACTGTATACACATAAGGAACGGGATTTCCTCAGAAAACATCATCTATATGATTAAAATAGCATTTTTAGAGTCAGAGATACAGATCATGCACACAAAACTAAATAGTTCTGAACAGAAAGCATCCTGTGGCCTTACCTGCAACGCTCTTTTTTCTCCAGTAAAAACACGAGCATCTAATTCATGTTGCATCTTTTCGGCAGCATGAATGGCTTGAAGAAAATCCATAACCAACTTTCCATCTTTTGTTATGTAACCTTCAGCAGATTCCTCGGTGCTACCCTGCAATTTCAACGAACCAAGGTTTATTGAACTCAAAGATTTATGATGTTGACAAAGTACTAAAGTAGTCAAAATTT

>band18

TAGGTAAGAAAATATCTACTCTAACTATTTAATAGGATCTGCTAGCTAAACTAGGATAGGACTAGAACTAATATGGTAACTATCTAACTAGCAAATAAACTAATAAGATCTGACTGTATACAAATAAGGAACGGGATTTCCTCAACAACACCACCACCACTGCAATCCTCGAATACAAATCTACAAAAGGAAAAACCTCTCCGGTTCTGCCTCAGCTTCCGGCCTTCAACGACACTAACACTGCAAGGACATTCACTTCCCAAGTCAGGAGCCTTACATCCAATGTGAATGTACCCAAAAAGATCGATAAATCCTTGTTCTTCACCGTGGGGCTAGGGTTGAACAATTGTACCAAACTGAACAGCCCTCGTTGCCAAGGTCCAAACGGCACCAGATTCACCGCAAGCATCAACAATGTGTCGTTCGTGTTTCCCAGGAG

>band_19

TAGGTAAGAAAATATCTACTCTAACTATTTAATAGGATCTGCTAGCTAAACTAGGATAGGACTAGAACTAACATGGTAACTATCTAACTAGCAAATAAACTAATAAGATCTGACTGTATACAAATAAGGAACGGGATTTCCTCACCTATAGTAATAGGATCTCGTCATCTTCTTGTCTGATCTAAATGCAGGATTTGCCTCGTACTTTTCCTGGCCATCCTGCCCTGGATATTGATGGCAGAAATGCTCTTAGGCGGATACTTACAGCCTATGCACGGCATAACCCCTCAGTTGGATACTGCCAGGTACTTGACTTTAAACTTCATAGCAGGGGAAGATATTTCTGTTATTCCTAGGCCAANNTGACTTTTAACATTGTTCTCATTATTGCTCTGCCATATATTCAGGTCAT

>band_21

TAGGTAAGAAAATATCTACTCTAACTATTTAATAGGATCTGCTAGCTAAACTAGGATAGGACTAGAACTAATATGGTAACTATCTAACTAGCAAATAAACTAATAAGATCTGACTGTATACACATAAGGAACGGGATTTCCTCAAATACGTTGGTCATTGCTGTTTTTATAACTCTTGCAGTATCCAAGCACTGCACAAGAAAGTGCTCCTTGGAGAAAATTATCAGGTGGAAGGAAATGGTAAAGATATCATGGATGATAATTGGAATCATGATCAGGAAATTCGTTGTGCAATCTGGAGGCTAGTTGGCATTTGTAGTTCAGATGACTCAGGAAGCATCAGAGCCTTGGTTTCTGATTTTGTATCTCGGGTATTATTCTTGCT

>band_22

TAGGTAAGAAAATATCTACTCTAACTATTTAATAGGATCTGCTAGCTAAACTAGGATAGGACTAGAACTAATATGGTAACTATCTAACTAGCAAATAAACTAATAAGATCTGACTATATACAAATAAGGAACGGGATTTCCTCAGTAAATCACACATGTCAGGCTAGTTACATTCTTCTTTTTTAGTATGAAAATGAAAGCCAAAGACAGTATGTATCACTTACGGCTGAGACAAGGTTATCATCCCACTTAACCATTGAAGAGAAAGAAGCAGAAGTTCATGGAGCTCTTTTGCAGGAAGGTCTTTCTCCACTGCTTCAGCGTGCTTCAGGAAAAACAAGCCGGCCTGTAAAAGGCAAACAAAGAGG

>band_23

TAGGTAAGAAAATATCTACTCTAACTATTTAATAGGATCTGCTAGCTAAACTAGGATAGGACTAGAACTAATATGGTAACTATCTAACTAGCAAATAAACTAATAAGATCTGACTGTATACAAATAAGGAACGGGATTTCCTCACCTCTCAAAGTGCATAAACTTCAGTAAATTTTGACATTGAACCAGTAAGAAATTTACACAAGTTTCTTCACCACAAAAAACACTCTGAAGTCGCAGCAATCTGCAAGAAGACAAACACAGATTGAATAAGCATATGAAGGAGTAATAACTAATATAGATAGAGAAATAGCACAACCAAATAACTTGTCAAACAAACAATGGG

>band_24

TAGGTAAGAAAATATCTACTCTAACTATTTAATAGGATCTGCTAGCTAAACTAGGATAGGACTAGAACTAACATGGTAACTATCTAACTAGCAAATAAACTAATAAGATCTGACTGTATACAAATAAGGAACGGGATTTCCTCAAAAATCGATATCCGCTGTGCCATCCAAGGTGATGTTGTGCTTGAATGTGTCAGCATACGGGATGAAATGGAATCTGAGGAAATGATGTTTCGGGTAGTGTTCAATACAGCTTTCATCAGGTCAAACATCTTGATACTCAATCGAGATGAAATTGACATATTATGGGATGCTAAAGATCTATTCCCAAAG

>band_25

TAGGTAAGAAAATATCTACTCTAACTAATTAATAGGATCTGCTAGTTAAACTAGGATAGGACTAGAACTAATATGGTAACTATCTAACTAGCAAATAAACTAATAAGATCTGACTGTATACACATAAGGAACGGGATTTCCTCAGCATCTTTTTTCATCCTAAATGGTGTAGATGGCATTCCCCTAGTCAAAGATAATGTTTCTACTTTCTACTGTCAGTAGTCTAGTGAAA

>band_26

TAGGTAAGAAAATATCTACTCTAACTATTTAATAGGATCTGCTAGCTAAACTAGGATAGGACTAGAACTAACATGGTAACTATCTAACTAGCAAATAAACTAATAAGATCTGACTGTATACAAATAAGGAACGGGATTTCCTCAATATCTAATAACAGGAGCATAAAGTACATGTCCAAATTTATTTGAAATAAAAAATCCTTCTATC

**RLC_Lu1-primer2 * EcoRI**

>RLC_Lu1-1 (LTR_sense)

TGAGGAAATCCCGTTCCTTATTTGTATACAGTCAGATCTTATTAGTTTATTTGCTAGTTAGATAGTTACCATATTAGTTCTAGTCCTATCCTAGTTTAGCTAGCAGATCCTATTAAATAGTTAGAGTAGATATTTTCTTACCTAGTCAGTAGGAGGAATGCTGTATATAATAAACCTACGAGACATGAATAAAAGTAATTCATTCTCTCAATCTTCA

>RLC_Lu1-1 (LTR_reverse)

TGAAGATTGAGAGAATGAATTACTTTTATTCATGTCTCGTAGGTTTATTATATACAGCATTCCTCCTACTGACTAGGTAAGAAAATATCTACTCTAACTATTTAATAGGATCTGCTAGCTAAACTAGGATAGGACTAGAACTAATATGGTAACTATCTAACTAGCAAATAAACTAATAAGATCTGACTGTATACAAATAAGGAACGGGATTTCCTCA

>band_9

AACGGGATTTCCTCACTTTCTGTTTCTGTGCGGTGTTGACATATTGTTATATTTGTAAATTTTTCAAGGTCCAAAGTATGAAGAGAAATATTGGAAGATTTTCTGGCTTCGTTTGGACTGGGAATGAGGTTTGGATCTGTTAAGTTATACATTTAATGTAGTTTTTTAGCTGAAATTCTCCTATGAAGTGTATGTTGCAACTCCATGTGTGGACACAAGATTTACCTTAGGACTTTAGTGACCATTTTGTGAAGCGTTTTTTGAATTGTAACTTTGATTCTGGTTGACAACTCTTGCTTGCTTGACAGGAAAAACAAAAGTCAAGAATGAAGGAAAAGCTTGACAAGTGTGTTAAGGAAAGTCTTCTAGACTTCTGTGATGTACTCAATATTCAAGTAACTAAAGCCACCGTGAGAAAGGTCAGTTGTGACTTCTAATGTTAATATATTCGCCGGGTTATCCTTAAATTCATCTTTTAGCTCGATAATGTTTTCAGGAAGATCTCACTGTAAAAATCTTGGAGTTCTTGGAATCTCCTCATGCAACAACTGATGTTATGCTTGCTGACAAGGAACAGGTATTCAATACTTGAATGAGTTTGCTGCAGTTTGGGTAGTTGGGGTTTCTTTTTCAATGCTATTACCTAGCTGATTTTGGATCTTATATTCGGTTTCAGAAAGTCAAGAGGCGGAGGTCAATGACTGGGAAAAATTCAAGCCCTGGGGAAGCATCAGCTACACTAGCTAAGGTTAGAATTACTGTAGTTAATGGACAACATAGATGTTCTGCAACTGTCAGAAATTTCCTCAATGCAGTGATCTCAATACAAGTAGCCCTGATAGTAGCAGTAAA

>band_12

AACGGGATTTCCTCACATGCTTTTATTTAACTAATTATACGGCATTCATTAAACTTCCATTCAGTTCTGCTTGCATGCTACTTGATAACATAAGATGTAAAACAGGAAACGGAGGAATTGTTTTGAAGAACTGAAGTGAAAAACGAAGACAAGTGAAGACCATGGGCCACTTACCAGAAAGGTGCTTCACATCAACATCCGTAATGCAGTTGCACCAATTGAGGTTAAGAGATTCCAACTTTGACAAACCTACAAACAGCAGGACATTTTAATAGCCTAGAGAAAACAATGCAAGATACCAGAAACTGAATGATGCAAGAGAAAATTAGATAAGCAAGTAAGAGTTAGATAAAAGTGTGAAAATACAGAAATGGAAAGGTAAAGTGAGTACCGTTGAGATGAACAAGAGCACCACCGCCAATGCCAGGACCCCTCTCCATGTCCAATTGAACTAGGTTTTCCAATGTGGCAACCGCCCTCAATCCCTCCGCACCGATGGCATTATTTCGTCGGAAGCTCAATCTTACCAAGTTTGAGCAGCCTTCTGGAAGCAAACAATGGTATAAGAAAATCTTCAACTCCCCAAAAAAATGTCACAATCATAACAAGCCAAATGGNTAATATCTATGAGCCGCAGA

>band_14

AACGGGATTTCCTCAGTATCGGATCCTAGTTGTAGAAGGAGAAAAAATGTTCACCCAGTGTTTTTGTGGTCAAAACAGCAATTGTTCCCAAAGCCCTGAATTTGCCTCTGCTAGCCAAAACGGTATGGCTGCGTGGATGGGTACTAGACAAAGGTCACTTCTAGTTGATGTAGATAATTCTTTAATAATTTTTGAAAATGACCTTTATATGACTTCCGACTTCTGTGTTCTAGCTTAATATAATGCAACATGTGATAAGCAGCTTAGTTTTTTTACCTACTTCCATTCACCAAAGTGTTCTACTGAAAGTCTGAAACAAGTTAGCAAACATCGTTTAAACTGAAGCCAGTAAGAAATGTTATTATTCTCCTTTGTGCACGTGGGTTTGTTGACTGCACTGGCTTTGTATGGCCTGCAGTTGAATGGGCTACAGGTAGGCTTATGGTTTCTCGGGTATTCAGGAACCAGTTATTTCTTTCATTCGTTTTGGCTGAATGACGACCAGA

>band_15

AACGGGATTTCCTCAGTAGTATGCCTGTCCTTTGGCTGACAGACATAGGAACCATAAATAGAGCCTCACTACATCAGTGACGGCTATTTCTTCATCATTTGAGCCAGAGAAACAAATTAGACTCATGAGAGTTCCTGTTTACAAATGCGGTTCTGTCCTTTGACTAACAGATGTATGAACCATTGAAGGTATTGTCACCATATCAATGACGGTACAAGCTCTTCAGCGTGGGAGCTAGGTGAAACAAATTTGATCTATTCATTTTCCCTTTTTTCTTTTGTTTATTTAAAAGCAGGATTGAAGCCTATGACAATCACACTTTCTTTAATTGCAGCGTTTAATAAATGTACTTTGTTTCACTTCTAAGCGATATTATATTCACTGTGTTTGGACATCTGAAAAGGAGACCAATATTAGAGCGGGCAACAAAAGCAAGATACTACCGGGA

**RLC_Lu2-primer1 * EcoRI**

>RLC_Lu2-1 (LTR_sense)

TGTTAAATGGTGTTAAATTATAAGTATGTGAGTGAGTTGTAATAGATAATAGTAGTAGTGGTATAAATAGAATTCTCCTTTCTGTTTGTACATTTAATTCATTCAGTACAGTAATAACAGAAAACGACTTTCCATTACAGCTTCTCCTTCTTCTTCGCTTTCTCTGTTCTTCTCTTCTCTTCCATTCATCAAACTTCACA

> RLC_Lu2-1 (LTR_reverse)

TGTGAAGTTTGATGAATGGAAGAGAAGAGAAGAACAGAGAAAGCGAAGAAGAAGGAGAAGCTGTAATGGAAAGTCGTTTTCTGTTATTACTGTACTGAATGAATTAAATGTACAAACAGAAAGGAGAATTCTATTTATACCACTACTACTATTATCTATTACAACTCACTCACATACTTATAATTTAACACCATTTAACA

>band_2

TTCTTCTCTTCTCTTCCATTCATCAAACTTCACACCTTGGCGTACTCGAGGACGAAGTAGATCTTCGTTTTCGAGGCCAGGACTTCGTAGAGGTGCAACATGTTTTTGTGTTGGTGAACGAGTCTCATGATGTGGATCTCTTGCTTGATGTTGGTGGTGGTTAGTCCTGCTTTCTGAGCCTTTTCCTTGTCGATTACCTTGATTGCTACACTGTTTCCGGTTTGGAGGTCTCGAGCGTAGTGGACTTTGGCGAATTGGCCTTGGCCTAGCAGCCTGCCGACTTCATACCTCTCCATCAAAATTTTGGTCCCATTGTTCTCCATTTCCAGTGAAGAGTTCTTTTTAACACACCGAATAGGTTCTACATGAGCTGGCAGGCTTCCATTACACGTTTCCTGCATGTCCTGGTGTTGCAGCACTACAGATAGATCTGGATCAAAGAAGTAAAGAGTTAGAAATCCATTAGTATGTACTGTCAGCCAAAAGGATCATCAGATTACAGGCATTTTGGTTGGATTCTGGTATAAAAACCAATCTGATTAACCCTCTCCCATCAACTCAAGTTATTTGAAAAACTCAAGTTATTTGAAAAAATTGAGATGCAATGNTTCTTAAGAGGTCTCCATTCT

>band_4

TTCTTCTCTTCTCTTCCATTCATCAAACTTCACATACTTAGCTGCAATCGTGAAAGAAACGTTGAGGCTACACCCGACGGGTCCTTTACTAATCCCCCACCGGTCCATGGGGACTTCAGAAGTGATGAATTACACGATCCCCGAGGAGGCCTTGGTCGTCGTTAATATGTGGGCGATCTCTCGAGATTGTTCGATCTGGGGGGATGATGCGTTGTCGTTTAGGCCGGAGAGGTTCGTCGGTTCGAAGGTAGATTTTCGAGGACAAGATTTCGAGTTGTTGCCATTTGGTGCAGGGAGGAGGATGTGTCCCGGGATGCCATTGGCGGCAAGGCAAATTCCTCTGCTCTTGGCTAATTTGGTTTGGAACTTTGATTGGTGCTTGCCAGACGGAGGAGATCCAGCGGTGGAGTTGGATATGAGCGAGAAGTTTGGGCTCATTTTACACAAGGAGCGGCCTCTGGTTCTTGTTCCTCGTCCGAGTTCTATATTACAAGACTGATGAGTAAGTGTCTTCTTCAAGTGTAGTAGAGTTATGTTGGTAATTTTAAGATATCCTATTT

>band_5

TTCTTCTCTTCTCTTCCATTCATCAAACTTCACACCATCCTCAACTGCTCTCACCCAACTCTCCTCCTCGTCAGAGAATCGGCTTTCCAAGACCCAATCAAAGCTCACAAGCTCCAATCCCTCACCAATGCCGGCGCCTCCGTCATTAAGGTGTTATTCAATTGGGATAATGCTTTTTCCAACTAGGGGAATTTTTGTTGTTCTTGATTCTTCTTCTTCTGCTGCTGTATTTTTAGGTTTCATTGCAGGACGAGAGTAGCCTTGTGGAGGCAGTGAATCGAGTTGATGTTGTGATCTGTTCGATTCCGTCTAAACAAGCTCATGATCAGAAGCTGCTTATCAATGTCATCAAACAAGCCGGAAGCCGGATAAAGGTAACTGTTAGATTGTGATTGTCAAATTGCTGTCTTCTTTGATTATCAAAAGCCGGATCAAGGCAACTGATTTTAGTGAAATTGAAGCGATTTCTTTGATGGGATTTTGTACATTAGCACATTTAATGTTAACATGGGTTTCATTCATGATGATCATCATTTTCCAGAGGTTCATCCCGTCC

>band_6

TTCTTCTCTCTTCCATTCATCAAACTTCACACATTTGACCATTTCTCGGTCCTACTTCCTATCAACATGTCTCGTTGGCGGTTCCCTCTACAGATTGTTTTGTGTTGTTGGTCGGCTTTTTTGCCTGTTATGTCACCCCAAAAATTAGGGATCTGCAATTTTAAAACTGCTCTCATCCTTATCTCTTCACCCGTCACTAACTGCGATTCTTTTCCACTCGTGTTTATTTCCAAGTTTGCATCACTATCCATCTTTCCCTTTTGCCCGATATCGCCTTCACCGATTCCAAATTCTGTCTCTGACTATCTCATTCTTTACTTGGATTTCTTATTCCCTCCAGATTCACTTGATATGGATTCTCTGCCACCAGCCATTATCCCTCATGGATTCGATGTGGAGTTTACGACGAAGGACGTGCGTGACGTTCCTCTAATTACACAACTCAGCCTTATTGGGTGATTTTTATGGCCTCTTCCCAAACCGACACATACCCTTCTCCAAGGAATGGCTCGAGTTTGGA

>band_7

TTCTTCTCTTCTCTTCCATTCATCAAACTTCACAGAAACAACAACCCAACTGATTTCCATTTCGACGAAGTCGCAGACGAGCAGGAGGACCTCGTCGCCGTTCAGTTTCCGCCGCGGTTTCGCGTCCTCGTCTAATTCCGCTTCTCTGCGGAGTTTACAGGTTTCCGATTCTCCCGTTATCTTACTTGAGCTTAATCGGAGCTACCCGTCTCTGTATCTATGTGCTAAATAGTATTATGCAACTTTTGTTTCTTGTGGGGGCAGTTTCAAATAACGGAGAAGATCTAATATAAGCGTGTGGTTGGAAAATTGATGAATTGAGGTACACGATGAGCAAGCAAAAGTCGAGTAGCTGTGGAATCTGTGAGAATACGAATCGTGCTTCCGTTTGTGCAGTTTGTGTCAATTACAGGTGAAATCTGCCTGACCCAACTTTTTTACGTTCTCTGGCTGGGAAAGTTGAAATTTATCACTTACGCTGTTTGTTTCATTGCAGGCTGAATCGCA

>band_9

TTCTTCTCTTCTCTTCCATTCATCAAACTTCACATGAGTGTCCTCTAAAATTATGTACACGTACTCTCCTTTCCCTTGCTTATGCAACTTGCAACATATATTTTAGCAAAGTTTTTAGAAGCTAGTTCTTTTTTGTCTTACTTTAGTATTGGATTTAAGGTATTTTGATTCTATATCGGCTACCGCGTGTTTTCGTAGAGCAGTACAGTTTATATCAGGTTGAATTTAGTGCATTTTACATATGTATTTAAAAAATTAAAACTATGTTATTTCGATTGTCTGTATTGTGTGGTTTATGAACAATGGCTACTTTTAGTAGAAATTCT

>band_10

TTCTTCTCTTCTCTTCCATTCATCAAACTTCACAATGCAATCAACTCTACGATTTTTCTTTGAATTAAAAAAAAGCATAGGCCAAATGAGGTCATTATTCGGCACTATGTTAATCACAAATCAATTACAAATCTCCAATTTCCCGCAAACGACAATAAATTTATACTCATTTTCTTTTCGAATAAATAAAAATACCTTATAATACATGTATTGCCGTACACTCACATATTTAAAGGGACGATTGGCTTTGCAATTGTGACGAAAATTTGGCTTCCACCATAAA

>band_12

TCTTCTCTTCTCTTCCATTCTTCAAACTTCACAAAGGAATCAGAGTTCGTTAAGTGGCAGAATGATGTAACCAAAATAGCCAAAATAGCCATCAGTATCTGGTTTACATGTATTCGTCAATGCAGTCAGTGATGTTAGATTGTTTGTATGGACAGGTTAAAATGCAGTTCTTGTTGAAAGGTGAGGTTTCGCCACAAGAGGTGATCGATGATTCACGAAGACAAAAGTGTCGAGCAGGGTTAGGTACTGAATTGGNTCNNANTCTA

>band_13

TTCTTCTCTTCTCTTCCATTCATCAAACTTCACAATAGTTAGTTACCGTGTGTTGAATCTGAAGCTATTCATGTTGTGATGGCAAGAAGAAGGAGAAATCCTACAGAATAAACCCAACTTTTCAGGCTAATCTCCAGAAGCATATAATGACCGGGNGAGGCCACATGTTTCGCAGCTCGACTATTTATCGTCACGTTAGTGAATGGATTCAGATGTTCATGTAGNATGTTTTTGTGTCCT

**RLC_Lu6-primer3 * EcoRI**

>RLC_Lu6-1 (LTR_sense)

TGTTGGTCCCGGATAATTGATGTGCAACAAAAATATGTACCACCAATATTAAAGCACCACAAAATTAGTTGGATCTCTAACCAACTAATTGAATTCCCACCAATATTAACCCCCACCTCCAATTTAGTGCACATGGAAATTAGTTGGGAATTCAACTAATTGGATGCCACAACCAATTGCTTTCAATTGGTCTCCCCACATTTCTAGTATAAAAGGGAAGCTAGTGCATCCCATTTCAATCATCCCTCTCTTCTTCCTTTCTCACTTCTCTAAGTGTTGTAGTGTAGCAATTTTCACTTGTTTAATAATTGAGATAAGTTATCTCAATTGGGTAGATAGGTGAGCGGTAGAAAGTCCCGGTAAATGTTTTACCGTGGTAGGAATACTTTCTTGTGAGCGATAAAATAGTGAGTAGTTGTTTCGGGGTTGGGAAACACTTGCGAGACACTATTTTGGATCGGCTCGGATCACCTTGTAGCTACCTTGTTATAGTGAAGAAGTGCTCGTAGCTGTCGCTGCTGCCGTAGATGTACTCTCCGCATTGGAGGGGAACTACGTAAATCCCGGTGTTATTTACTTACTGTTTTGTGCTTGGCAATTTCGAGAATATTCGTTGTATATTGCATTATTAATATTACCACAGTAAATTGGTCTAAGGAGGTTGGCTTAATTATCGTCATGATGGTATTGCGGTGGTAATCACCCATCCATAGTGATTTTAAGTGTGGCGGACTACCGCCACTTCCAACTTATCTGGGAAATATTTACGGTGTGTGGTTTATTAGTGCAATATATTTACTCTATTCTCGTCCGCTGCGCCCCAACA

> RLC_Lu6-1 (LTR_reverse)

TGTTGGGGCGCAGCGGACGAGAATAGAGTAAATATATTGCACTAATAAACCACACACCGTAAATATTTCCCAGATAAGTTGGAAGTGGCGGTAGTCCGCCACACTTAAAATCACTATGGATGGGTGATTACCACCGCAATACCATCATGACGATAATTAAGCCAACCTCCTTAGACCAATTTACTGTGGTAATATTAATAATGCAATATACAACGAATATTCTCGAAATTGCCAAGCACAAAACAGTAAGTAAATAACACCGGGATTTACGTAGTTCCCCTCCAATGCGGAGAGTACATCTACGGCAGCAGCGACAGCTACGAGCACTTCTTCACTATAACAAGGTAGCTACAAGGTGATCCGAGCCGATCCAAAATAGTGTCTCGCAAGTGTTTCCCAACCCCGAAACAACTACTCACTATTTTATCGCTCACAAGAAAGTATTCCTACCACGGTAAAACATTTACCGGGACTTTCTACCGCTCACCTATCTACCCAATTGAGATAACTTATCTCAATTATTAAACAAGTGAAAATTGCTACACTACAACACTTAGAGAAGTGAGAAAGGAAGAAGAGAGGGATGATTGAAATGGGATGCACTAGCTTCCCTTTTATACTAGAAATGTGGGGAGACCAATTGAAAGCAATTGGTTGTGGCATCCAATTAGTTGAATTCCCAACTAATTTCCATGTGCACTAAATTGGAGGTGGGGGTTAATATTGGTGGGAATTCAATTAGTTGGTTAGAGATCCAACTAATTTTGTGGTGCTTTAATATTGGTGGTACATATTTTTGTTGCACATCAATTATCCGGGACCAACA

>band_5

CCCATCTATAATGATTTTAAGTGTGGCGGACTACCGCCACTTTCAACCTATCTGAGATATATTTACGGTATATAGTTTATTAGTGCAATATATTTACTCTATTCTCGCCCGTTGCGCCTGAAAGACGTATTATTGTATCTATCACTTTTANTAACANAAGCATACAAAGATGCACATCAAAAGAGCGTCATGGTAATAAATGACACCTCCCTCTCAATCTTTGCTTGTTGGAATCTACGTTCCCTCCATTGACAGGAGCTACTCCTCCCCTGCCGCTGCTGCTGCGGCACATGTATCATCATCATCATGATCATAGTCACCCAGAAATAGTACATATGATGACGTTGACTTTCCATTN

>band_8

CCCATCCATAGTGATTTTAAGTGTGGCGGACTACCGCCACTTCCANCTTNCCTGGGAAATATTTACGGTGTGTGGTTTATTAGTGCAATATATTTACTCTATTCTCGTCCGCTGCGCCCCAACACATTGCTCTGTGTAATCTTCAACATAACTTCAATTCACATGTACTTGAGTTTCAAAATTTAAGACGACATCATCTACATACACCATTAGCATTACGAGAACCC

>band_9

CCTATCCATAGTGATTTTAAGTGTGACTGACTTCCGTCACTTCCAACCTACCTGTGAAATATTTATGGTGTATGATTTATTAGTGCAATATATATACTCTATTCTCGTCCGCTGCGCCCAACAGTAACCGATACATTTATCTTTGGTGGTGAGAGAACGACTTCAAGAAATTAACTAAGACCCGAATTCAACATTACCTTACCACGACAAAAATG

>band_13

CCCATCCATAGTGATTTTAATTGTGGCGGACTACCGCCACTTCCAACTTACCTGGGAAATATTTACGGTGTGTGGTTTATTAGTGCAATATATTTACTCTATTCTCGTCCGCTGCGCCCCAACAGGTTGGTCCCTTGAGNGNCGNGANNGCCTTGCTNNNGGCCGAGGTTGTGTTAAGACT

>band_15

CCCATCCATAGTGATTTTAAGTGTGGCGGACTACCGCCACTTCCAACTTATCTGGGAAATATTTACGGTGTGTGGTTTATTAGTGCAATATATTTACTCTATTCTCGTCCGCTGCGCCCCAACAGACGGGCGCCACCATCCTCCTACTCAAACTCACANCCTCGCT

>band_16

CCCATCCATAGTGATTTTAAGTGTGGCGGACTACCGCCACTTCCAACTTACCTGGGAAATATTTACGGTGTGTGGTTTATTAGTGCAATATATTTACTCTATTCTCGTCCGCTGCGCCCCAACAGTAACCTCTTCTACGGNGATTCTGAGTCCTCTTN

**RLC_Lu8-primer1 * EcoRI**

>RLC_Lu8-1 (LTR_sense)

TATTGGAAATGATTTTTCATTTTCCCGCCAAACTTCACACTCTCCAAGCTTCAAGTGAAACGGAGCGTTTCTTCCTCTCTACACCAACGACTAAATGAAACGGAGCGTTCTGTTAAGTGATGAAGATAAGAACAAAACGTCATCGTTGCACTGATCAGAACAGCTCATCGTCCTCTTCTCTGTTCCTCTGCATTTCTGCAAATTCCGTTAGAGCCTCAAGCTCACCTACTCTCTTTCAGCCAGCTACCAGCTGTGCACATTGTCTTTTAGCTTCTCATCACTTTGTATATGTACCACCTTTCTATCAATGAGAACGTTGAGCCATTTCATTTGAACACAAACGAGTTAATA

> RLC_Lu8-1 (LTR_reverse) TATTAACTCGTTTGTGTTCAAATGAAATGGCTCAACGTTCTCATTGATAGAAAGGTGGTACATATACAAAGTGATGAGAAGCTAAAAGACAATGTGCACAGCTGGTAGCTGGCTGAAAGAGAGTAGGTGAGCTTGAGGCTCTAACGGAATTTGCAGAAATGCAGAGGAACAGAGAAGAGGACGATGAGCTGTTCTGATCAGTGCAACGATGACGTTTTGTTCTTATCTTCATCACTTAACAGAACGCTCCGTTTCATTTAGTCGTTGGTGTAGAGAGGAAGAAACGCTCCGTTTCACTTGAAGCTTGGAGAGTGTGAAGTTTGGCGGGAAAATGAAAAATCATTTCCAATA

>band_4

GCACTGATCAGAACAGCTCATCGTCCTCTTCTCTGTTCCTCTGCATTTCTGCAAATTCCGTTAGAGCCTCAAGCTCACCTACTCTCTTTCAGCCAGCTACCAGCTGTGCACATTGTCTTTTAGCTTCTCATCACTTTGTATATGTACCACCTTTCTATCAATGAGAACGTTGAGCCATTTCATTTGAACACAAACGAGTTAATACAATCAAACTGGTTAAGATTACATACATGAGCCTGCTTTTTTGTCCACCTATTCATTCGGACATGTTAATGAATTAAAGAGTTGTGAAGAAATCAGCCCGTGATAAAAACTGAAAATGTGAATAATTGCGTAAGGCTCAGATCTGCCATTTGTTAATCAGACGGCGGTGGTCCAAGGTAGCAAGCCCGCATGATCACCGGCAGTTGATGGCTGCGAAATCGGAGCAGCTAGCTAGTGCTGCCTCGATCGCCACGCCCAACAGCCAGACCGAGTGAACTGGGACCCTCCACCTCAAAAATCGCCGTCCGAGTAAAAACTCTCCGGACGTATCATCACAGCCTATGTTCCCAGAAGAAGTAAGAAGAAGAAGCAGAAGAAGAAGTAGTATATATGATTAATTCCATGGAAACAGAGACAGACAGAGATAGATCATAGATAGATAGATATATAGCTAGCTAGTAGTAGTAGTAGTGTTAGCAGCAGCAGCAGTGAGGGTGGTGATGGTGGAATATAATGGATGGCGACGGCGGGTGTTGGGTGCCGGAGAAATCATCGGCACTCACAGCCTCATCCTCGAACGAGAGGGTGGCAGAGAACCTTTATATTAAATTAATTATACTTGGCTTTCTGTAGTTACAGGATTGCCCTTTTGTTCTCGTCTTGTCTGTGCCAGTCCACAGGAGAAAAAAGAGGTGGTGGTAAACTAGTAATTAGAATGAACATGAGAGGGTATTTCCGTGTAAGCC

>band_5

TAAGAACAAAACGTCATCGTTGCACTGATCAGAACAGCTCATCGTCCTCTTCTCTGTTCCTCTGCATTTCTGCAAATTCCGTTAGAGCCTCAAGCTCACCTACTCTCTTTCAGCCAGCTACCAGCTGTGCACATTGTCTTTTAGCTTCTCATCACTTTGTATATGTACCACCTTTCTATCAATGAGAACGTTGAGCCATTTCATTTGAACACAAACGAGTTAATAAGTGAGCTGATTCAGGAAGCGGTTTCTGCTGCTAGGGGTGAGCCCTCTGATGAGAATTTGGTGAGTTTGTTGAGTTTGATGTATGGGTACAGTTCGTTTAGGGATGGGCAACTTGAAGCTATTAAAATGGTGCTTGATGGGAAATCGACCATGTTGATTTTGCCCACTGGAGCTGGAAAATCACTTTGCTATCAAATTCCTGCCGTTATTTTGCCTGGGATACTTTAGTAGTAAGCCCGTTAGTCGCATTGATGATTGATCAGCTTAAACGGTTGCCTCCAGAGATTCAGGGTGGTCTTTTCTGTAGCAGTCAGGTAGTTTTCTCTCTTTATCTCTCTCTTCAATGGCCTTTTGCGTGTACCATCATGCTGTTTTGTTTAATGTAGCTTCTTTTGAGTTCCATAGACGCCTGAGGACGTTGCGGAACAATCAGGCAGCTTCAGCAAGGAGCCATTAGGGTAAGCTAGGTTAAGGTTTAGTTATAAGAAAAATCTATCTGTTTCCTGTGTAGTTGGGATGTTTGGTGAAAATTTATTGTTTGTTCCAGGTGCTATTTGTTTCGCCAGAGAGGTTCCTGAACGCAGATTTCTTGTCGCTTTTGTCTGAGATTCCTGTTTCCCTTCTGGTGGTCGATGAAGCTCACTGTATCTCTGAATGGTGACTTTCCTGTTGCTATGGTCCTCGCATTTATAAATGGGAGATGGGGTTTGTCATTG

>band_6

TAAGAACAAAACGTCATCGTTGCACTGATCAGAACAGCTCATCGTCCTCTTCTCTGTTCCTCTGCATTTCTGCAAATTCCGTTAGAGCCTCAAGCTCACCTACTCTCTTTCAGCCAGCTACCAGCTGTGCACATTGTCTTTTAGCTTCTCATCACTTTGTATATGTACCACCTTTCTATCAATGAGAACGTTGAGCCATTTCATTTGAACACAAACGAGTTAATACACCGCCGCCCGTCTGAAGGTGGTCGAGGGAAGCAGAGCAGGCCGTCGCGAGATGATAATGATGATGATGATGATGATCAGAAGGCTCCGTCCATGTCGATCGACGCTGAAGCAGAGTCAGCCGTCGGCGAATCCAATAATGGTTTATTCGAGGATGATATCGACAGATGTCCTACATTGCATTGCAAAATTCCCGCAGGTAATGACTGATACATACTAGTTGCTTGTTTCAGTGAGTTGTTTTCTGATTGTTGTTTCATATACAGGGAACGTTGTAAAAAATACAGGGAACTGGCACTTTGCTGGAGGACAGGGTTGTGATGGTAATGAACAAAATCAATGATCATTTTTAATTATGATATTATTTCATTATCCATAAACAATAATTTTGAAGGTTAATATTCTTATTCTGGTTTTTTCCCGACGGAATCGCTTGTATTAGTTGTAATGCCAAAGGCAGAGATCGAAGAGACGGAGAAGCTGGAGCGATTCAAAATCAAGCCCCGGAATTTCATGAATGTCGGAACCACCAAGTAATGTGTCCACAGTATGTAACACTCCGATTTTTCCGATAAAATATTGATCGATTTGGCCATAATTAAGCTCTTATAGATTTGTTTTCGAGTGCATAATAATGACTTCTCAAGAGATCACCCATTTACACTGTTACTCTCTGAATNTGTTTAACTTCANAT

>band_7

TAAGAACAAAACGTCATCGTTGCACTGATCAGAAAGCTCATCGTCCTCTTCTCTGTTCCTCTGCATTTCTGCAAATTCCGTTAGAGCCTCAAGCTCACCTACTCTCTTTCAGCCAGCTACCAGCTGTGCACATTGTCTTTTAGCTTCTCATCACTTTGTATATGTACCACCTTTCTATCAATGAGAACGTTGAGCCATTTCATTTGAACACAAACGAGTTAATACACGTTGACTTCCTAGTCTATGGTTACTCGTTACGTGTCACGGATTCAAAATGATCCAAAGTTCTATATCGAAATCCTAACCGTTGATCAATTCTGACAGATTCGACCGGGATTTGAGATGGAGGACGGGAGTGCAGCTGGTACCAATCGAATGCCAGAGCTCCAACAACTTCAAGCTGAGGATTGAAGATTTGGAATCTGCTTACGACATGGCTAAACTCAACAACGTTCGGGTCAAAGGATTGCTCTTGACCAACCCATCCAACCCGCTGGGTACAATTCTCGACGGGAACACTCTGAGAAGCATTGTCTCCTTCACCAACGACAACAACATTCACCTCATCTGCGACGAGATCTACTCCGCCACCGTCTTCGACAAGCCTGATTACGTCAGCGTCGCCGAGGTCGTCGACGAATACCTGAACAACGCTAACGACGATGGCGAGGGTGATGATGGTAATAGTAACGGACCCAGGCCCACTCTGAATCTGGACCTGATTCATATAGTGTACAGCCTCTCAAAGGACATGGGCTTCCCGGGTTTCCGGGTCGGGATAATTTACTCATACAACGACGTCGTAGTCAGCTGCGCAAGGAAGATGTCGAGTTTCGGTTTGGTTTCGACCCAGACCCAGCACCTGATCGGGTCGATGCTCTCTGACNATGATTTCGTCGACTAT

>band_9

TAAGAACAAAACGTCATCGTTGCACTGATCAGAACAGCTCATCGTCCTCTTCTCTGTTCCTCTGCATTTCTGCAAATTCCGTTAGAGCCTCAAGCTCACCTACTCTCTTTCAGCCAGCTACCAGCTGTGCACATTGTCTTTTAGCTTCTCATCACTTTGTATATGTACCACCTTTCTATCAATGAGAACGTTGAGCCATTTCATTTGAACACAAACGAGTTAATATGTGGGTCAGTCAAATTACTCAAATTTATGAGTTCACTGTGCTTTGATTTTCTTTCATTCTGTTAGTTTCTGGGTTTCTCACTGTTTGGTTGATTGAGTTCATCTCCAAAATCGTTGCTTGTTAGATTCCCCCCCTGCATGATTTGTGCATTTTCACCAGTGGTGGTTGATTCATCACTTTTTAACAAGTAGGTGCAATAGCATGAACAGTTTCATGCAGGCTCCAAAATATCCCCCATTTTTGCTGTGAGTTCATATGATCTTGTAGAGAGGGGTTTTCATCTCAGTGCCAAGTTGCATGTCAGATTCCCTCGACTCCGCCAAAAAACAATTGTGTGTTGATAACTCGAATTGTTAGGAGAGAGTTAAAGATGGATTTTTATATTACTTAACAATACGCCTTTTGTTCCCCTCAAACGGAAGGTTCTTAGAATAGTTGATTGTGTGCACAGTTTTGACAAGTCATGTGCTCCGAATGTGCTCCGAATTAGGGCTACGG

>band_10

TAAGAACAAAACGTCATCGTTGCACTGATCAGAACAGCTCATCGTCCTCTTCTCTGTTCCTCTGCATTTCTGCAAATTCCGTTAGAGCCTCAAGCTCACCTACTCTCTTTCAGCCAGCTACCAGCTGTGCACATTGTCTTTTAGCTTCTCATCACTTTGTATATGTACCACCTTTCTATCAATGAGAACGTTGAGCCATTTCATTTGAACACAAACGAGTTAATAGTATTTGTCGGGTTGTTTCACGTTGACCCTCGGTGGGAAGTAGCAGAAGCGGTTCTCCTCCACGTCGGTCTTCATTCTTCTGATTTCGGAGTCGGCTTATTTCTGAGGGTCTGCAGCAGGGCGATTTCCGGGTAGTGGCCGTTCGCGAATGGTGGTTTCGTTCATATAATAATCCCAACACTTCTTCAACAACTTTTCTTCGTTTTCGAACACATCTTTCTGTGAAAACGACCCACTAATTCCTGCAAATACCACGTCCTTACAGTACAGAAGATACGATGTCAAGTCTTCGGAAAACTCACATGGTCCTCCAATCTTGCTATACTCTGTTTCAAGTTTGTGCAGAAATTGCTCAGAGTCGAGCTCTGACGTATGAGTCGTCGGTGCACACTTGGATAAGCTCCTTTCTCATGCACCAAAACCATGCAATTTATAGA

>band_11

TAAGAACAAAACGTCATCGTTGCACTGATCAGAACAGCTCATCGTCCTCTTCTCTGTTCCTCTGCATTTCTGCAAATTCCGTTAGAGCCTCAAGCTCACCTACTCTCTTTCAGCCAGCTACCAGCTGTGCACATTGTCTTTTAGCTTCTCATCACTTTGTATATGTACCACCTTTCTATCAATGAGAACGTCGAGCCATTTCATTTGAACACAAACGAGTTAATACGGCTACTGCGGATCATGATATGATGACGAGGATCACGAGCGGTGATCACGTGGCGGACCTGCAGGATCAGTTCGAGAGCGGTGACGATGATGATGCGAACGTGGGTGAGTTTGGTTATAGTCTTGATGATGACGAGGATGATGACGTGGACGACGGTAATTTTATTCGTGGTAGTAATAATAATAATGATAATAATAACACTGGATTTGGGGATGACTTCTCTTCTTGGATATTCGGTAATAGCTCTTCCACCGGCGGCGGGGGGTGTTAGTGTAATTTTGCTTTTTGGTTCCTGATTTACTTTGTAGGTTAGGTAATTAGGTGAAAAGTGTGTAGAATTTCATGTAAAAATAATTCATTCCTCG

>band_12

TAAGAACAAAACGTCATCGTTGCACTGATCAGAACAGCTCATCGTCCTCTTCTCTGTTCCTCTGCATTTCTGCAAATTCCGTTAGAGCCTCAAGCTCACCTACTCTCTTTCAGCCAGCTACCAGCTGTGCACATTGTCTTTTAGCTTCTCATCACTTTGTATATGTACCACCTTTCTATCAATGAGAACGTTGAGCCATTTCATTTGAACACAAACGAGTTAATACTCGGCTTTTCACAAGCTTGATGGCTAGCTCTCCGCTCCTCGAAACCCTAGAAATCATCCGTTATGTTTACGGGATGAGGAACCTAAATTTTCCCAATCTGAAGACCCTCAAAATTTCAACAATCATCGACAGGGACAAGAGTACTGATGGATTGTTCATGGACGAGTTCATAGCCCCTCAGCTGAATACTCTGGAAATTGATAATTGTTTTTATTTGAGATTGAGTGATGTATCTCGGGCAGTTTCTAAGCTCGAGAATCTGAAGTACTTGACCCTTACTCGATTCGATCCACCAGAGAAGACACTGAAACTTTCGTGTCCCAAGCTCGAG

>band_13

CGTTGCACTGATCAGAACAGCTCATCGTCCTCTTCTCTGTTCCTCTGCATTTCTGCAAATTCCGTTAGAGCCTCAAGCTCACCTACTCTCTTTCAGCCAGCTACCAGCTGTGCACATTGTCTTTTAGCTTCTCATCACTTTGTATATGTACCACCTTTCTATCAATGAGAACGTTGAGCCATTTCATTTGAACACAAACGAGTTAATAGATCCTAAGCTGGATCCTGCCACCCTTTTATTCAACGACTTTTCTGTTTGATTCTTCTTCTTCCTCCTCCTCTCTATTTATGGTTATGTCATCTCAATTTGTTTCGTAATTGAATTTGATCAGAGTCGTCAGTCTTTACACTCATTTCACTACTTTTTCAGTTTATTTCCCGTAATTTTTCCTTTTAAAAAAAACTGGATGAATGGAGGGTAATTTGGCCAATGCACAATTTACTCATTTTTTTCGGTGTTGCTATTCATTTAATGAAATTTTGAAAGTTGTTACACGATGATTACAG

>band_14

TAAGAACAAAACGTCATCGTTGCACTGATCAGAACAGCTCATCGTCCTCTTCTCTGTTCCTCTGCATTTCTGCAAATTCCGTTAGAGCCTCAAGCTCACCTACTCTCTTTCAGCCAGCTACCAGCTGTGCACATTGTCTTTTAGCTTCTCATCACTTTGTATATGTACCACCTTTCTATCAATGAGAACGTTGAGCCATTTCATTTGAACACAAACGAGTTAATACACAACATTCTTCGTAAGAAATTCCTAGCTTTGCAAAAGAAAACCAAAAAAAGTTTTAATGCTGCTAGCTATTACTCCGTAGAAGCATTTCACATGTTAAAAAGGAACTTACCTGAAAGGAAAGCAAGTGACGTGGAATTGAAGAGATAGGTAGTCCACACAGAAGCCTAGGAAATGAACAATTTAGCTGCTAAAGAATTGACAGAAAACTGAAGGCAGAGGCACGACA

>band_15

TAAGAACAAAACGTCATCGTTGCACTGATCAGAACAGCTCATCGTCCTCTTCTCTGTTCCTCTGCATTTCTGCAAATTCCGTTAGAGCCTCAAGCTCACCTACTCTCTTTCAGCCAGCTACCAGCTGTGCACATTGTCTTTTAGCTTCTCATCACTTTGTATATGTACCACCTTTCTATCAATGAGAACGTTGAGCCATTTCATTTGAACACAAACGAGTTAATAATAGATACGGCGGTTTTGCTTCAAGGAAGTCTAATGTTAGGAATTGGGCTGATTCGGATGCTAAGCCGGCAAAAGATTATTACTTTGACGGCCATGGTGATCGGGATAATTTAGCTTATGGCTCACTCTACAGGTTTTCGCTTTTGCTTTGTTTACACTGGAAATTATGCAGGATTT

>band_16

TAAGAACAAAACGTCATCGTTGCACTGATCAGAACAGCTCATCGTCCTCTTCTCTGTTCCTCTGCATTTCTGCAAATTCCGTTAGAGCCTCAAGCTCACCTACTCTCTTTCAGCCAGCTACCAGCTGTGCACATTGTCTTTTAGCTTCTCATCACTTTGTATATGTACCACCTTTCTATCAATGAGAACGTTGAGCCATTTCATTTGAACACAAACGAGTTAATAACTATCAACCACAAGGACCAGGCCAAGTGTGTTGCGAATTGCGATGCGATGTCTATCTGAATCGAGATATTTTTCCTACACTAATTATAATTAATTAAGTAGATTTAAACAAGAATTTCAGTTCCAATTATTAGAAGCATT

>band_17

TAAGAACAAAACGTCATCGTTGCACTGATCAGAACAGCTCATCGTCCTCTTCTCTGTTCCTCTGCATTTCTGCAAATTCCGTTAGAGCCTCAAGCTCACCTACTCTCTTTCAGCCAGCTACCAGCTGTGCACATTGTCTTTTAGCTTCTCATCACTTTGTATATGTACCACCTTTCTATCAATGAGAACGTTGAGCCATTTCATTTGAACACAAACGAGTTAATACTTTTTCCTGCCACTGGTAACCCGTAGAATGGCCTGGAAACGCGGCGAATGCATTTCCTTGGCAAACGCCGCATGACGACTCTACAGATAATTGGACA

>band_18

TAAGAACAAAACGTCATCGTTGCACTGATCAGAACAGCTCATCGTCCTCTTCTCTGTTCCTCTGCATTTCTGCAAATTCCGTTAGAGCCTCAAGCTCACCTACTCTCTTTCAGCCAGCTACCAGCTGTGCACATTGTCTTTTAGCTTCTCATCACTTTGTATATGTACCACCTTTCTATCAATGAGAACGTCGAGCCATTTCATTTGAACACAAACGAGTTAATAAACTGCGAGAGCTTGGAGCATCTATTTTTGCAGGA

**RLC_Lu28-primer1 * EcoRI**

>RLC_Lu28-1 (LTR_sense)

TGTTGAAAAATATTATATTTTCCTTATTAGTATAGGAATAAGTTTCAATATTTTTCCTTATTAGTATAGGAATAGTAATAAGAGTTTTCCTAGTTGAGGAAGGATTCTCCTATCCTAACTCTATATAAACCCATGTACCCCTTATGTAATCTCATATATCATAATACCATTGAAAACTTCCTCTCATAAATTCAATA

> RLC_Lu28-1 (LTR_reverse)

TATTGAATTTATGAGAGGAAGTTTTCAATGGTATTATGATATATGAGATTACATAAGGGGTACATGGGTTTATATAGAGTTAGGATAGGAGAATCCTTCCTCAACTAGGAAAACTCTTATTACTATTCCTATACTAATAAGGAAAAATATTGAAACTTATTCCTATACTAATAAGGAAAATATAATATTTTTCAACA

>band_1

CCATATATCATAATACTATTGAAAAGCTTCCTCTCATAAATTCAATAAAGATCAACGGCATACTCCCCAAAGTTTGAGTGACCAAGTAACCGGGCATACTTGTGCCGCAGTTCAACCTAAAAGAAGAAAGGAAAGATTCTCTTCAGGATACTACTTTAACAAAAGGTAACCAGGTCATATGCTTCATCATATCATTCCAAAGTTACATCAGTAAGTAGCAACACCAATCAAACACGGACTGCTCATGATAACTGACAACAACCTTGAGCTACTATTTCCTTGAATCAATGCTACTTATTTCTGAATCTTGATAGTTAGCTTTTTTATTTTTCTACTTTTCGGTCCGTATGTTCTTTCTTTGTTAGGATAGGGAGTATATATAGAGCATAAACGACTGCAGGTACCTTTAGCCAACAGCCGACTCGAATGTATAAGTAAACAAGGTTTAATAAAATCATCACTTGTTAGAAGTGTTCAAACAGTTTAGTAATTTCTTCAAAAGTTTAATACATAAACCCAAAATAGGAATAAGGTCACCGAAGGACGATATCATCCTAGGAATCTATTGAAACTTAAATGAATTGCTCTAACCGGTCAAACATTAAAGAATCTTACCAAACTTTCGAAAACTGAAAGATTGATATGTCCACATCTCTTCCCGTATGCAACCGCAACTCGCTTTCTGGTTTGTCCTACCTGTATACATTAAGAAACATAACACGGCCTTAACCCACCATTATCCAAA

>band_4

CCATATATCATAATACTATTGAAAAGCTTCCTCTCATAAATTCAATATATACTTTACATATTGATTTGTTCTTCAAACGTTAAACACGTGAATGATGCTAGCTAATGTCTGCTGCTGCTAACTCTGTTATGCAGTGGTTTGGAGATGCTGAGAAGTTGACAAAGGCCCTTTTCTCCTTTGCCACCAAGCTTTCTCCTGTTATCATATTTATCGACGAGGTACCTCCCTAAACTCTCTTTTTCACTAGGGCTTTGTCAATCGACATGTTCGACTTAACATGTTGTGGTTGGCGGGGGCGGAAAGAAAAGGTAGATAGTCTACTAGGGGCTCGAGGGGGTTCTCACGAGCACGAGGCTNCGAGAAGAATGAGAAACGAGTTCATGGCAGCATGGGACGGATTAAGATCGAAATACACTCAAAGGATCGTCATCCTTGGTGCCACGAATCGNCCGTATGATCTTGATGATGCTGTGANTCGNCGTTTNCCTAGGAGGTAAGGCGCGCNTGGAACNCTCTCTTTGTCGTCCGANGANTTTTATAAATGCCNTCGGAAANNCANCTGTGCTCCTNGTTTAGAATATNCGCG

>band_6

TCATATATCATAATACCATTGAAAACTTCCTCTCATAAATTCAATANATACCAAAAGATCAACTGNTNTGTTGACAAATTGCACAATCTCANNCAGCAATGTAGACCGTCCATAAACAAAACCTNTGCAACAAACCACAAAGTAAACTCCACTTCATTAAACAAGCGAATCATCTTCACTTCGCTGGAACAACAAAAACCACCATCTCGCTCTTTCCTTTCTTCAGTGCTGCCTTTTTCTTTCCAAGTTGATGATCATCAGTTCTTGAAACACCAACCACACTTTGGTTGCTTAACTGCTCCAAAAACTTTCCTAACCCTTGAAATCTCCAGAAACAATTGGTTTGGCCATCATATCTTGAAAGATTCACAATTGAATCATGTTCAAAACAGTATATAATGCATCGATACTATCCGATGGACCATGAGAAATCGATTATAACCTGAAGAAGAAACTCAAAACTAATGTTTTCAGAACAATTGTACCTGAGATGGTGTTATGAGTTTCAAAACTAATGTATTCTAACTACCAACTATGATGAATCCAAGCTCTCTAAACGTTTAACACAACAAAAATTGTGTGCATTCTATATCTATCTC

>band_7

CCATATATCATAATACTATTGAAAAGCTTCCTCTCATAAATTCAATACATACCTTAAAACGAGCATGCAAGGTCAGCAAAATGTCATTCAGCAGAGCAGCTGGCCAAGCTGGATCAGAAAGCTCCGAAACAATGATTGATTCCAGCTCATCCAATGATTCATATGGAAGTTGCATCCATATCAATGCTTTGATCACCATTGCTCGAACATAAACACATTCACAGGCAACAGTTGTTCGAACAACCTCCATCAGTGATGCTAATAAACTTGCAATTGTTTCCTTGCTACCAGTTGAACTCGATAAAGAAGAAGCCTTTCGGGCACCTGAAATAACAATGAATCAATGAGAGGAAATGGGGGTACAAATGCGATGTAAAAAAAAATCAATACTGCAAAAGCCAGAAACTCCTAATTTCTACCGAATCAAATTCAATGTAAGAAGGCGGAACCATAGAAACCCAAAAATCTTGAGACCATGTGAATAAACTAGGAAACTCCTTATACTGCATAAAAGGAGTAACAAGTGAGGTTTC

>band_9

CCATATATCATAATACTATTGAAAAGCTTCCTCTCATAAATTCAATACAACTCNNACCCCCGCAGGNCCACANAAGNAATTTCNGGAAAAAAAAAATAGAATCTCTACCTCCTTTCCCATTTTCTCAACTTTACCTTCTGTTTCATGCATTCCACCTTTTGGTGTAATCAATCATAGGGAACCTTAAATCACTAATTAATTAACATGCTATGTGAATGATGGCTCTTCTTCAAGGCAAACATCCCATCTTTAGATGTTTCAAACACATTTAACCCATCGATGGTAATACCCTTTTAATGATAAGCTAGATTTTCAGAGTAAATAGGTATAATAGTAGCTCATAAGTTAGATCAAACCTTGACGTACAATACCTCATCCAATCAGGTAACGCGAGTGAATAGCCTAATGAGAGGCTAAGGAATGCTCGATCAAGGNGNAAAGANAAATTTTAGGATATTTTTATGTC

>band_11

CCATATATCATAATACTATTGAAAAGCTTCCTCTCATAAATTCAATAGTCTTGTTGCCATGCTTATTTGCATTTGACATCAAAAGCCAAATTTCCAGGCAAGGAGCTGTTGTTGACGAGGTTTTCCAAAATGTGTTGTCTCTTTTGAGCAAGAACTACACTTTAGTGACTGATGAGTTAGTTGGAGTTGATCATCATGTGGAAGAAGTGATGAAATTACTGAATTTGGGCTCAGGATGTGTGACGACTGTTGGCATTTATGGAATGGGCGGAATTGGAAAAACCACCATCGCTACAGCTGTCTATAACAAAGTCTGCACGCTTTTTGACCGTTGTAGCTTTGTTGATGATGTAAGAGAAACATTGTCATGGAGTGATGGTATTGTCACTTTGCAGAATAAGCTCATCTATGGCATTACGAAAGATGGCTCTCCCATTGGTAGCACAAGTGAAG

>band_12

TCATATATCATAATACCATTGAAAACTTCCTCTCATAAATTCAATACTTCCACTCATGCGACAGTAGCTGCTGAATCTTCTAAGTTATAACTGAAACCAATAGTGTATAACTTGCATAATAAACAGAAAAGTCTACGAGAAGGAAAAAATTAACCTACGTCAGTCTAAGGTGTAGCCTGAACCTAGTTTATATATACAAACTAATATAGGACGATATATCATACAAGAGATCTTAGCCCCTTCTCCACCAGCAATCTATATATCGAAAACTGTCCAGGCTGCATACCTTTGCCATTAATATTGTCAACTGAAATATCTTATGAGAAATATGTAAATCCCATCTCTTCGAGAGCTGGAGAATAGATTTTGCAGCAGCTAATCGGATATAAGGCTTGTCACTTGCACTGCCCAAAG

>band_14

TCATATATCATAATACCATTGAAAACTTCCTCTCATAAATTCAATAATTTATCATCTCTAGTCAAAAGAACATTGAAATGTAATTCGTATAGGGAGTTCATATATGCCGTACTGAAATACTTGTCGTAGTTAATCCGGGTACCGTAGTTACGTTGTCTTTCCAGTGAACAAGAGTTTCCATTAACCGCCGCTATTAAAATCTGTCGACATCCCAGATGGTTCAAGTGTTTTAAGAGTTT

>band_20

CCATATATCATAATACTATTGAAAAGCTTTCTCTCATAAATTCAATACATTCAAATGTCGCTTAAGCTGAAATTTGAAGTATGATTGTTCCAGATGCACAATGTGCGGTGGAAA

>band_22

CTATATATCATAATACTATTGAAAGCTTCCTCTCATAAATTCAATAGTTCCTTAAAATCCCCTGCAGCGTCTACTTTGGTGGCCCATTCAATTTTTTCAGCTGCACT
