# Supplementary material for: Ty1-copia elements reveal diverse insertion sites linked to polymorphisms among flax (Linum usitatissimum L.) accessions
Source: BMC Genomics. 2016 Dec 7;17:1002. doi: 10.1186/s12864-016-3337-3 (PMC5142383; doi:10.1186/s12864-016-3337-3)
Supplement: Additional file 6: — Comparison of selected SSAP band scores and PCR validation in 14 flax accessions. SSAP band polymorphisms were selected for validation using conventional PCR (see Methods). 1 = present, 0 = absent, W = weak band at expected size, (?) = weak band at non-expected size, 1+L = expected band plus an additional lower band, 1+H = expected band plus an additional higher band, P = polymorphic among replicates of same cultivar. Colors from accessions represent flax types as in Fig. 3. (DOCX 41 kb) [file 12864_2016_3337_MOESM6_ESM.docx]

**Additional file 6. Comparison of selected SSAP band scores and PCR validation in 14 flax accessions. SSAP band polymorphisms were selected for validation using conventional PCR (see Methods). 1 = present, 0 = absent, W = weak band at expected size, (?) = weak band at non-expected size, 1+L = expected band plus an additional lower band, 1+H = expected band plus an additional higher band, P = polymorphic among replicates of same cultivar. Colors from accessions represent flax types as in Figure 3.**

|  | | | **OS** | | | **OW** | | | **FW** | | **FS** | | | | | |  |
| --- | --- | --- | --- | --- | --- | --- | --- | --- | --- | --- | --- | --- | --- | --- | --- | --- | --- |
| **Copia family primer** | **Band ID** | **Technique** | **rdf** | **bet** | **lut** | **ole** | **bli** | **oli** | **vio** | **ade** | **sci** | **eve** | **dra** | **her** | **bel** | **aur** | **Match^2^** |
| **LTR_RLC_Lu0-primer3** | 7 | SSAP | 0 | 0 | 1 | 0 | 0 | 0 | 0 | 0 | 0 | 0 | 0 | 0 | 0 | 0 |  |
|  |  | PCR | 0 | 0 | 1 | 0 | 0 | 0 | 0 | 0 | 0 | 0 | 0 | 0 | 0 | 0 | PERFECT |
|  | 8 | SSAP | 0 | 0 | 0 | 1 | 0 | 0 | 1 | 0 | 0 | 0 | 0 | 0 | 0 | 0 |  |
|  |  | PCR | 0 | W | 0 | 1 | 0 | 0 | 1 | W | 0 | W | W | W | W | W | NO |
|  | 12 | SSAP | 0 | 0 | 1 | 0 | 0 | 0 | 0 | 0 | 0 | 0 | 0 | 0 | 0 | 0 |  |
|  |  | PCR | 0 | 0 | 1 | 0 | 0 | 0 | 0 | 0 | 0 | 0 | 0 | 0 | 0 | 0 | PERFECT |
|  | 17 | SSAP | 0 | 0 | 0 | 0 | 1 | 1 | 0 | 0 | 0 | 0 | 0 | 0 | 0 | 0 |  |
|  |  | PCR | 0 | 0 | 0 | 0 | 1 | 1 | P | 0 | 0 | 0 | 0 | 0 | 0 | 0 | NEARLY PERFECT |
| **LTR-RLC_Lu1-primer1** | 18 | SSAP  PCR | 0  0 | 0  0 | 0  0 | 0  0 | 0  0 | 0  0 | 1  1 | 0  0 | 1  1 | 1  1 | 1  1 | 1  1 | 1  1 | 0  0 |  |
|  |  |  |  |  |  |  |  |  |  |  |  |  |  |  |  |  | PERFECT |
|  | 19 | SSAP | 1 | P | 0 | 0 | 0 | 0 | 0 | 1 | 1 | 1 | P | 0 | 0 | P |  |
|  |  | PCR | 1 | 1 | 0 | 0 | 0 | 0 | 0 | 1 | 1 | 1 | 1 | 1 | ? | 1 | NEARLY PERFECT |
|  | 23 | SSAP | 0 | 0 | 0 | 0 | 0 | 0 | 0 | 0 | 1 | 0 | 0 | 0 | 0 | 0 |  |
|  |  | PCR | 0 | 0 | 0 | 0 | 0 | 0 | 0 | 0 | 1 | 0 | 0 | 0 | 0 | 0 | PERFECT |
|  | 26 | SSAP | 0 | 0 | 0 | 0 | 1 | 1 | 1 | 0 | 0 | 0 | 0 | 0 | 0 | 0 |  |
|  |  | PCR | 0 | 0 | 0 | 0 | 1 | 1 | 1 | 0 | 0 | 0 | 0 | 0 | 0 | 0 | PERFECT |
| **LTR-RLC_Lu1-primer2** | 9 | SSAP | 0 | 0 | 0 | 1 | 0 | 0 | 0 | 1 | 0 | 0 | 0 | 0 | 0 | 0 |  |
|  |  | PCR | L | L | L | 1+L | L | L | L | L | L | L | L | L | L | L | NO |
|  | 12 | SSAP | 1 | 1 | 1 | 0 | 1 | 0 | 1 | 0 | 0 | 1 | 0 | 1 | 0 | 1 |  |
|  |  | PCR | 1 | 1 | 1 | 0 | 1 | 0 | 1 | 0 | 0 | 0 | 0 | 1 | 0 | 0 | NO |
|  | 16^1^ | SSAP | 0 | 0 | 0 | 0 | P | 1 | 1 | 0 | 0 | 0 | 0 | 0 | 0 | 0 |  |
|  |  | PCR | 0 | 0 | 0 | 0 | P | 1 | 1 | 0 | 0 | 0 | 0 | 0 | 0 | 0 | PERFECT |
|  | 18^1^ | SSAP | 0 | 0 | 0 | 1 | 0 | 1 | 0 | 0 | 0 | 0 | 0 | 0 | 0 | 0 |  |
|  |  | PCR | 0 | 0 | 0 | 1 | 0 | 0 | 0 | 0 | 0 | 0 | 0 | 0 | 0 | 0 | NEARLY PERFECT |
| **LTR-RLC_Lu2-primer1** | 2 | SSAP | 1 | 1 | 1 | 0 | 0 | 0 | 1 | 1 | 0 | 0 | 1 | 1 | 0 | 0 |  |
|  |  | PCR | 1 | 1 | P | 0 | 0 | 0 | 1 | 1 | 0 | 0 | 1 | 1 | 0 | 0 | PERFECT |
|  | 7 | SSAP | 0 | 0 | 0 | 1 | 1 | 1 | 1 | 0 | 0 | 0 | 0 | 0 | 0 | 0 |  |
|  |  | PCR | 0 | 0 | 0 | 1 | 1 | 1 | 1 | 0 | 0 | 0 | 0 | 0 | 0 | 0 | PERFECT |
|  | 10 | SSAP | 0 | 0 | 0 | 0 | 0 | 0 | 0 | 1 | 1 | 1 | 1 | 1 | 1 | 1 |  |
|  |  | PCR | 0 | 0 | 0 | 0 | 0 | 0 | 0 | 1 | 1 | 1 | 1 | 1 | 1 | 1 | PERFECT |
|  | 13 | SSAP | 1 | 1 | 0 | 0 | 0 | 0 | 0 | 1 | 1 | 0 | 1 | 0 | 1 | 0 |  |
|  |  | PCR | P | W | 0 | 0 | 0 | 0 | 0 | 1 | P | 0 | 1 | 0 | 1 | 0 | PERFECT |
| **LTR-RLC_Lu6-primer3** | 5 | SSAP | 1 | P | 0 | 0 | 0 | 0 | 0 | 0 | 0 | 1 | 1 | 1 | 1 | 1 |  |
|  |  | PCR | 1 | ? | P | ? | 0 | 1 | P | 0 | P | 1 | 1 | 1 | 1 | 1 | NO |
|  | 8 | SSAP | 1 | 1 | 0 | 1 | 1 | 0 | 0 | 1 | 1 | 1 | 1 | 1 | 1 | 1 |  |
|  |  | PCR | 1 | 1 | H | 1+H | 1+H | H | H | 1+H | 1 | 1 | 1 | 1 | 1 | 1 | NO |
|  | 9 | SSAP | 1 | 1 | 0 | 1 | 1 | 1 | 1 | 1 | 1 | 1 | 1 | 1 | 1 | 1 |  |
|  |  | PCR | 1 | 1 | 1 | 1 | 1 | 1 | 1 | 1 | 1 | 1 | 1 | 1 | 1 | 1 | NO |
|  | 16 | SSAP | 1 | 1 | 1 | 0 | 0 | 0 | 1 | 1 | 1 | 1 | 1 | 1 | 1 | 1 |  |
|  |  | PCR | P | 1 | 1 | 1 | 1 | P | P | 1 | P | 1 | 1 | 1 | 1 | 1 | NO |
| **LTR-RLC_Lu8-primer1** | 7 | SSAP | 0 | 0 | 0 | 1 | 1 | 1 | 1 | 0 | 0 | 0 | 0 | 0 | 1 | 0 |  |
|  |  | PCR | 0 | 0 | 0 | 1 | 0 | l | 0 | 0 | 0 | 0 | 0 | 0 | W | 0 | NO |
|  | 15 | SSAP | 0 | 0 | 0 | 0 | 0 | 1 | 1 | 1 | 0 | 0 | 0 | 0 | 0 | 0 |  |
|  |  | PCR | 0 | 0 | 0 | 0 | 0 | 1 | 1 | 1 | 0 | ? | ? | ? | ? | ? | NEARLY PERFECT |
|  | 17 | SSAP | 0 | 0 | 0 | 0 | 0 | 0 | 0 | 1 | 0 | 0 | 1 | 0 | 0 | 0 |  |
|  |  | PCR | 0 | 0 | 0 | 0 | 0 | 0 | 0 | 1 | 0 | 0 | 1 | 0 | 0 | 0 | PERFECT |
|  | 18 | SSAP | 0 | 0 | 1 | 0 | 0 | 0 | 0 | 0 | 0 | 0 | 0 | 0 | 0 | 0 |  |
|  |  | PCR | 0 | 0 | 1 | 0 | 0 | 0 | 0 | 0 | 0 | 0 | 0 | 0 | 0 | 0 | PERFECT |
| **LTR-RLC_Lu28-primer1** | 11 | SSAP | 0 | 0 | 0 | 1 | 0 | 1 | 1 | 0 | 1 | 0 | 0 | 0 | 0 | 0 |  |
|  |  | PCR | ? | ? | ? | 1 | ? | 1 | 1 | ? | 1 | ? | ? | ? | ? | ? | NEARLY PERFECT |
|  | 12 | SSAP | 0 | 0 | 1 | 0 | 0 | 0 | 1 | 1 | 1 | 1 | 1 | 1 | 1 | 1 |  |
|  |  | PCR | 0 | 0 | 1 | 0 | 0 | 0 | 1 | 1 | 1 | 1 | 1 | 1 | 1 | 1 | PERFECT |
|  | 14 | SSAP | 1 | 1 | 1 | 0 | 0 | 0 | 0 | 0 | 0 | 0 | 0 | 0 | 0 | 0 |  |
|  |  | PCR | 1 | 1 | 1 | W | W | 0 | 0 | W | 0 | W | W | W | W | W | NO |
|  | 20 | SSAP | 0 | 0 | 1 | 1 | 0 | 0 | 0 | 0 | 0 | 0 | 0 | 0 | 0 | 0 |  |
|  |  | PCR | 0 | 0 | 1 | 1 | 0 | 0 | 0 | 0 | 0 | 0 | 0 | 0 | 0 | 0 | PERFECT |

^1^These two bands are not displayed in Table 3 or Additional file 4 since they were redundant to bands 13 and 15 from LTR-RLC_Lu1-primer1 respectively.

^2^Nearly perfect matches are defined as only having one mismatch, or perfect matches with additional weak bands at a different size from the expected band.
